# cDNA Microarray Gene Expression Profiling of Hedgehog Signaling Pathway Inhibition in Human Colon Cancer Cells

**DOI:** 10.1371/journal.pone.0013054

**Published:** 2010-10-01

**Authors:** Ting Shi, Tapati Mazumdar, Jennifer DeVecchio, Zhong-Hui Duan, Akwasi Agyeman, Mohammad Aziz, Janet A. Houghton

**Affiliations:** 1 Department of Cancer Biology, Lerner Research Institute, Cleveland Clinic Foundation, Cleveland, Ohio, United States of America; 2 Department of Computer Science, University of Akron, Akron, Ohio, United States of America; University of Hong Kong, China

## Abstract

**Background:**

Hedgehog (HH) signaling plays a critical role in normal cellular processes, in normal mammalian gastrointestinal development and differentiation, and in oncogenesis and maintenance of the malignant phenotype in a variety of human cancers. Increasing evidence further implicates the involvement of HH signaling in oncogenesis and metastatic behavior of colon cancers. However, genomic approaches to elucidate the role of HH signaling in cancers in general are lacking, and data derived on HH signaling in colon cancer is extremely limited.

**Methodology/Principal Findings:**

To identify unique downstream targets of the GLI genes, the transcriptional regulators of HH signaling, in the context of colon carcinoma, we employed a small molecule inhibitor of both GLI1 and GLI2, GANT61, in two human colon cancer cell lines, HT29 and GC3/c1. Cell cycle analysis demonstrated accumulation of GANT61-treated cells at the G1/S boundary. cDNA microarray gene expression profiling of 18,401 genes identified Differentially Expressed Genes (DEGs) both common and unique to HT29 and GC3/c1. Analyses using GenomeStudio (statistics), Matlab (heat map), Ingenuity (canonical pathway analysis), or by qRT-PCR, identified p21^Cip1^ (CDKN1A) and p15^Ink4b^ (CDKN2B), which play a role in the G1/S checkpoint, as up-regulated genes at the G1/S boundary. Genes that determine further cell cycle progression at G1/S including E2F2, CYCLIN E2 (CCNE2), CDC25A and CDK2, and genes that regulate passage of cells through G2/M (CYCLIN A2 [CCNA2], CDC25C, CYCLIN B2 [CCNB2], CDC20 and CDC2 [CDK1], were down-regulated. In addition, novel genes involved in stress response, DNA damage response, DNA replication and DNA repair were identified following inhibition of HH signaling.

**Conclusions/Significance:**

This study identifies genes that are involved in HH-dependent cellular proliferation in colon cancer cells, and following its inhibition, genes that regulate cell cycle progression and events downstream of the G1/S boundary.

## Introduction

Hedgehog (HH) signaling plays a critical role in a variety of normal cellular processes. It is pivotal in embryogenesis, regulation of the epithelial-to-mesenchymal transition, the patterning of a diverse range of vertebrate structures in a variety of organs, maintenance of adult tissue homeostasis, tissue repair, cellular proliferation, and in cell survival [1], [2], [3], [4], [5], [6], [7], [8], [9]. The canonical HH pathway is also critical to normal mammalian gastrointestinal development, where it is involved in the coordinate regulation of differentiation of normal intestinal villi [10], [11], [12]. Thus, in the normal gastrointestinal tract, HH ligands are induced in the differentiated cells around the villous surface, generating a negative feedback loop to inhibit canonical WNT signaling in the basal cells of the crypt, thereby protecting differentiated cells from the proliferative effects of WNT [13]. Activation of the canonical HH signaling pathway comprises the binding of HH ligands to the membrane receptor Patched (PTCH1), which becomes internalized leading to the activation of the signaling molecule Smoothened (SMO) via release from PTC-mediated suppression. SMO activates the final arbiter of HH signaling, the GLI family of transcription factors that bind to the GACCACCCA-like consensus binding element in promoter sequences to transcriptionally regulate HH target genes [3], [14], [15]. GLI1 and GLI2, the transcriptional activators of HH signaling, possess distinct as well as overlapping functions that involve activator (GLI1 and GLI2) or repressor (GLI2) activities [16]; however, their roles in the regulation of HH-driven cellular proliferation, survival or cell death processes are poorly understood. Historically, GLI1 has been considered the most reliable marker of HH pathway activity, however GLI2 appears to be the primary activator of HH signaling, with GLI1 as a transcriptional target of GLI2 [3], [7], leading to augmentation of HH signaling both quantitatively as well as qualitatively [16]. An important feature of GLI proteins is that their biological activity is context-dependent, influenced by the cellular environment [17], [18].

Activation of the canonical HH signaling cascade is aberrantly activated and well known to play a critical role in oncogenesis and maintenance of the malignant phenotype in several types of human cancers. Such activation involves amplification of GLI1 or GLI2, mutations in PTC or SMO, or dysregulated gene expression [3], [4]; these malignant cells are also sensitive to the small molecule inhibitor that targets SMO, cyclopamine [4], [19], [20], [21], [22], [23]. Colon carcinomas are thought to derive from constitutive activation of WNT signaling by mutation of the APC or β-CATENIN genes, while the involvement of the HH signaling pathway is not as clear. In gastrointestinal malignancies, transcriptional up-regulation of HH ligands has been identified as the predominant activator of HH signaling in these diseases (reviewed in [3]). In addition, there is emerging evidence that HH signaling is involved in colorectal carcinogenesis [24], [25], colon carcinoma stem cell self renewal, and in the metastatic behavior of advanced colon cancers [26]. However, genomic approaches to elucidate the role of HH signaling in cancers in general are lacking, regulatory genes downstream of GLI1 and GLI2 that function in cellular proliferation, survival, and maintenance of the malignant HH phenotype remain incompletely characterized [5], and data derived on HH signaling in colon cancer is extremely limited.

Cellular proliferation is driven by progression of cells through the cell cycle consisting of sequential passage through G1, S, G2 and M phases. Cyclin-dependent kinases (CDKs) associate with cyclins to drive the cell cycle machinery [27], [28]. Thus, CDK2 associates with CYCLIN E at the G1/S transition and with CYCLIN A during S-phase, CDK4 and CDK6 bind to CYCLIN D during progression at G1/S, while CDC2 complexes with CYCLIN A at G2, and with CYCLIN B during the G2/M transition. CDC25 family members also regulate cell cycle progression through dephosphorylation of the CDKs [29], [30]. CDK inhibitors, including p21^Cip1^ [29], [31] and p15^Ink4b^ [[32], bind to cyclin-CDK complexes during the cell cycle transition, in particular at G1/S (p21^Cip1^, p15^Ink4b^;[32]) and G2/M (p21^Cip1^;[29]), and can also induce cell cycle arrest at the G1/S boundary following cytostatic signals through functional inhibition of cyclin-CDK complexes. The E2F family of transcription factors also regulates the expression of genes required for the G1/S transition, in particular genes involved in the activation of the DNA replication machinery, and DNA repair [33].

cDNA microarray technology has provided the ability to study the expression of thousands of genes simultaneously, and is an important tool in the dissection of signal transduction pathways. For the HH signaling cascade, HH/GLI target gene expression has been examined following EGF stimulation [6] or inducible GLI1 [16] or GLI2 [9] gene activation in human keratinocytes, or in GLI1-induced cell transformation [7]. To identify unique downstream targets of the GLI genes that function in cellular proliferation in the context of colon carcinoma, we employed a small molecule inhibitor of both GLI1 and GLI2, GANT61, identified in a cell-based small molecule screen for inhibitors of GLI1-mediated transcription [34]. GANT61 acts in the nucleus to block GLI1 function, inhibits both GLI1- and GLI2- mediated transcription, and demonstrates a high degree of selectivity for HH/GLI signaling [34]. Thus, GANT61 acts downstream of cyclopamine (targeting SMO) to inhibit the final determinants of HH transcriptional regulation.

In two human colon carcinoma cell lines, HT29 and GC3/c1, inhibiting the HH signaling pathway using GANT61 decreased expression of GLI1, GLI2 and the HH ligand receptor, PTCH1, and inhibited proliferation by inducing cellular accumulation at the G1/S boundary 24 hr after treatment, determined by flow cytometric analysis. On further detailed analysis using cDNA microarray gene expression profiling and quantitative Real-Time PCR, p21^Cip1^ (CDKN1A) and p15^Ink4b^ (CDKN2B), that can elicit the G1/S checkpoint, were up-regulated, while genes that further determine entry from G1 to S-phase including E2F2, CYCLIN E2 (CCNE2), CDC25A and CDK2 were decreased in expression. Concomitant with decreased G1 to S-phase progression, decreased expression of CYCLIN A2 (CCNA2), CDC25C, CYCLIN B2 (CCNB2), CDC20 and CDC2 (CDK1), that regulate the passage of cells through G2/M were also demonstrated. Additional novel genes that are involved in stress response, and the response to DNA damage, not previously identified following termination of HH signaling in human cancer cells, include the early response genes DDIT2 (GADD45G), DDIT3 (GADD153), DDIT4 (REDD1), PPP1R15A (GADD34) and ATF3 that were significantly up-regulated. Genes involved in DNA synthesis and repair (TYMS, TOP2A, TK1, POLE, POLE2), and additional novel genes involved in S-phase progression or DNA damage responses that were significantly down-regulated, include KIAA0101 (p15 [PAF]), Replication Factor C variants 2, 3, 4, 5, CDT1, the E2F transcription factors CDCA4 and TFDP1, MDC1, FANCD2, PCNA, and the genes involved in DNA repair, RAD51C (XRCC3), RAD54B, RAD51 and HELLS. This study has therefore identified genes that are regulated during the termination of HH-dependent cellular proliferation and survival in colon cancer cells, and involves genes associated with G1/S-phase arrest, DNA damage and stress responses.

## Results

### GANT61 induces down-regulated expression of GLI genes and accumulation of human colon carcinoma cells at G1/S

**Figure 1:**
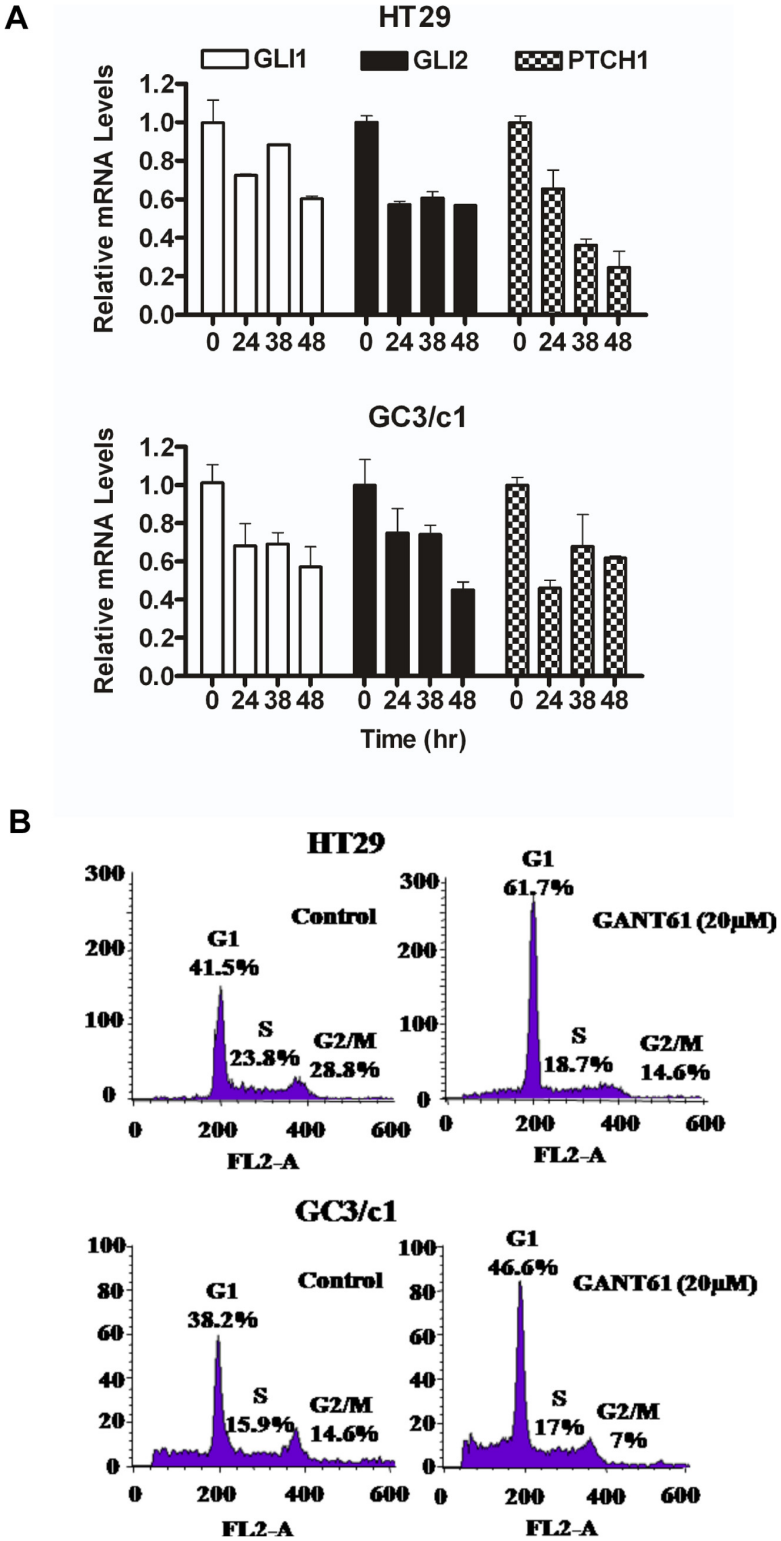
Expression of GLI1, GLI2, PTCH1 and cell cycle distribution of HT29 and GC3/c1 cells. Cells were treated for up to 48 hr with GANT61 (20 µM) or with 0.2% DMSO (vehicle control). (A) qRT-PCR of GLI1, GLI2 and PTCH1 genes from time 0 to 48 hr. (B) DNA was extracted at 24 hr after treatment, stained with propidium iodide, and subsequently analyzed for the effects of inhibition of HH signaling on phase distribution of cells within the cell cycle by flow cytometry.

In HT29 and GC3/c1 cells treated with GANT61 (20 µM) for up to 48 hr, expression of the target genes GLI1 and GLI2 were both down-regulated, and also the HH ligand receptor PTCH1, as determined by qRT-PCR (Figure 1A). Subsequently, HT29 or GC3/c1 cells were treated, in duplicate, with GANT61 (20 µM) followed by PI staining and flow cytometric analysis for the determination of cell cycle distribution between G1, S and G2/M phases (Figure 1B). In both cell lines, cells accumulated in G1, 24 hr after treatment with GANT61. In GANT61-treated HT29 cells, a 20% increase in G1-phase cells was associated with a corresponding decrease in cells within the G2/M phase (14%) and in S-phase (5%), consistent with a G1/S checkpoint arrest. In GC3/c1 cells, an 8% increase in G1-phase cells at 24 hr after GANT61 treatment also corresponded with a similar reduction in cells in the G2/M phase of the cell cycle (Figure 1B).

### cDNA microarray analyses identify changes in gene expression both common and unique to HT29 and GC3/c1 following GANT61 treatment

**Figure 2:**
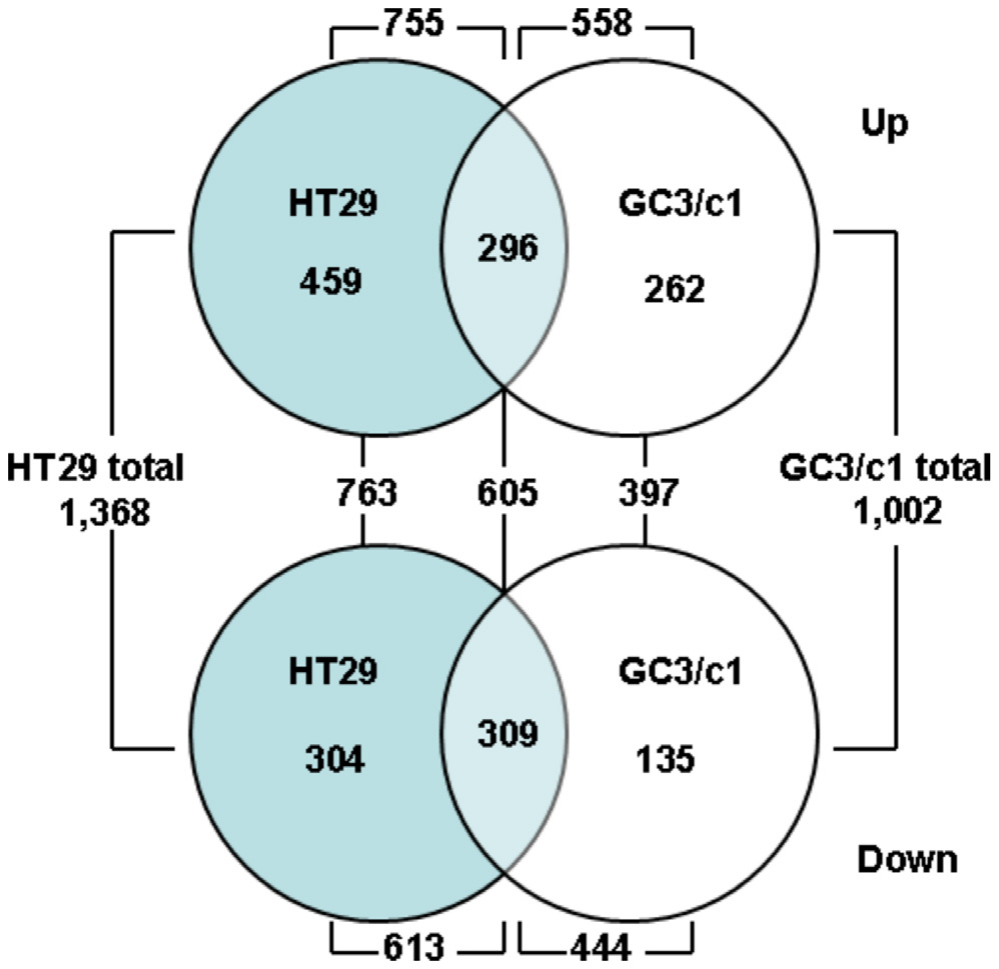
Venn diagram summarizing Differentially Expressed Genes (DEGs) in GANT61-treated HT29 and GC3/c1 cells. Cells were treated with GANT61 (20 µM) or vehicle alone (0.2% DMSO) for 24 hr, and total RNA extracted as described in Materials and Methods. Changes in gene expression were determined by cDNA microarray gene profiling using the Illumina Human-ref8 V3.0 Bead-Chip array. Genes with a False Discovery Rate (FDR)-adjusted p-value (p<0.001) and fold change >1.5 were considered DEGs. Upper panel: up-regulated genes. Lower panel: down-regulated genes. Differential expression for the total, unique up-regulated, unique down-regulated, common up-regulated, and common down-regulated DEGs, are shown.

To delineate the changes in gene expression in HT29 and GC3/c1 human colon carcinoma cell lines in response to treatment with the GLI1/GLI2 antagonist, GANT61, the expression of 18,401 human genes was profiled in control cells treated with vehicle (0.2% DMSO) and in cells treated with GANT61 (20 µM) for 24 hr. Genes with a False Discovery Rate (FDR)-adjusted p-value of <0.001 and fold change >1.5 were considered Differentially Expressed Genes (DEGs) induced by GANT61 relative to the vehicle control, of which 1,368 genes were differentially expressed in HT29, and 1,002 genes in GC3/c1 cells (Figure 2). 755 genes or 558 genes were up-regulated, and 613 or 444 genes were down-regulated, in HT29 and GC3/c1 cells, respectively. 763 and 397 genes were differentially expressed and unique to HT29 or GC3/c1, respectively. Of the 763 DEGs unique to HT29, 459 (60%) were up-regulated and 304 (40%) were down-regulated. Similarly, of the 397 DEGs unique to GC3/c1, 262 (66%) were up-regulated and 135 (34%), down-regulated. In contrast, 605 genes representing 3.4% of all genes were differentially expressed that were common to both cell lines; of these, 296 were up-regulated (49%), and 309 (51%) were down-regulated (Figure 2). All genes common to both HT29 and GC3/c1 that were significantly up-regulated or down-regulated (p<0.001) are listed in Tables 1 and 2, respectively.

**Table 1:** Up-regulated genes (p<0.001) common in GANT61-treated HT29 and GC3/c1 cells.

ACCESSION NUMBER	GENE SYMBOL	DEFINITION	Fold Change		ACCESSION NUMBER	GENE SYMBOL	DEFINITION	Fold Change	
			HT29	GC3/c1				HT29	GC3/c1
NM_080489.3	SDCBP2	syndecan binding protein	12.42	10.83	NM_001008213.1	OPTN	optineurin	2.63	2.37
NM_001042483.1	NUPR1	nuclear protein 1	7.96	3.53	NM_000431.1	MVK	mevalonate kinase	2.61	2.77
NM_021187.2	CYP4F11	cytochrome P450	7.73	4.27	NM_001550.2	IFRD1	interferon-related developmental regulator 1	2.60	1.84
NM_001001870.1	C17orf91	chromosome 17 open reading frame 91	6.79	3.82	NM_025130.2	HKDC1	hexokinase domain containing 1	2.58	4.46
NM_004864.1	GDF15	growth differentiation factor 15	6.61	3.43	NM_002461.1	MVD	mevalonate (diphospho) decarboxylase	2.57	5.36
NM_004083.4	DDIT3	DNA-damage-inducible transcript 3	6.36	2.35	NM_021626.1	SCPEP1	serine carboxypeptidase 1	2.55	1.79
NM_005130.3	FGFBP1	fibroblast growth factor binding protein 1	5.86	41.19	NM_012424.2	RPS6KC1	ribosomal protein S6 kinase	2.53	2.03
NM_022060.2	ABHD4	abhydrolase domain containing 4	5.65	3.07	NM_052839.2	PANX2	pannexin 2	2.53	5.61
NM_015526.1	CLIP3	CAP-GLY domain containing linker protein 3	5.25	2.74	NM_021173.2	POLD4	polymerase (DNA-directed)	2.51	1.71
NM_000389.2	CDKN1A	cyclin-dependent kinase inhibitor 1A	5.21	2.02	NM_020400.4	LPAR5	lysophosphatidic acid receptor 5	2.50	2.17
NM_002298.2	LCP1	lymphocyte cytosolic protein 1	5.02	8.60	NM_007075.3	WDR45	WD repeat domain 45	2.50	1.61
NM_019058.2	DDIT4	DNA-damage-inducible transcript 4	4.91	3.91	NM_001909.3	CTSD	cathepsin D	2.50	1.68
NM_031412.2	GABARAPL1	GABA(A) receptor-associated protein like 1	4.73	4.81	NM_022157.2	RRAGC	Ras-related GTP binding C	2.48	2.80
NM_033285.2	TP53INP1	tumor protein p53 inducible nuclear protein 1	4.71	2.80	NM_138573.2	NRG4	neuregulin 4	2.48	2.69
NM_001001342.1	BLOC1S2	biogenesis of lysosome-related organelles complex-1	4.68	3.95	NM_201545.1	LGALS8	lectin	2.46	2.81
NM_153282.1	HYAL1	hyaluronoglucosaminidase 1	4.57	22.29	NM_175738.3	RAB37	RAB37, member RAS oncogene family	2.40	3.53
NM_012385.1	P8	p8 protein	4.57	4.01	NM_012257.3	HBP1	HMG-box transcription factor 1	2.40	1.65
NM_016061.1	YPEL5	yippee-like 5	4.39	3.98	NM_001083617.1	RB1CC1	RB1-inducible coiled-coil 1	2.39	1.68
NM_145693.1	LPIN1	lipin 1	4.39	3.59	NM_153341.1	RNF19B	ring finger protein 19B	2.38	1.61
NM_019600.1	KIAA1370	KIAA1370	4.13	2.73	NM_003129.3	SQLE	squalene epoxidase	2.38	3.03
NM_021009.3	UBC	ubiquitin C	4.08	7.52	NM_018045.5	BSDC1	BSD domain containing 1	2.38	2.34
NM_002084.3	GPX3	glutathione peroxidase 3	4.05	2.99	NM_003376.4	VEGFA	vascular endothelial growth factor A	2.37	2.09
NM_003256.2	TIMP4	TIMP metallopeptidase inhibitor 4	4.04	2.02	NM_007028.3	TRIM31	tripartite motif-containing 31	2.37	6.54
NM_198336.1	INSIG1	insulin induced gene 1	4.02	9.90	NM_000527.2	LDLR	low density lipoprotein receptor	2.35	3.83
NM_002517.2	NPAS1	neuronal PAS domain protein 1	3.95	2.76	NM_024717.3	MCTP1	multiple C2 domains, transmembrane 1	2.33	2.28
NM_001040619.1	ATF3	activating transcription factor 3	3.90	1.86	NM_198833.1	SERPINB8	serpin peptidase inhibitor	2.32	2.07
NM_006096.2	NDRG1	N-myc downstream regulated gene 1	3.89	2.63	NM_023039.2	ANKRA2	ankyrin repeat, family A (RFXANK-like)	2.31	1.82
NM_003670.1	BHLHB2	basic helix-loop-helix domain containing	3.82	3.68	NM_025047.1	ARL14	ADP-ribosylation factor-like 14	2.30	6.15
NM_002130.6	HMGCS1	3-hydroxy-3-methylglutaryl-Coenzyme A synthase 1	3.75	6.54	NM_001080391.1	SP100	SP100 nuclear antigen	2.29	1.90
NR_003086.1	HSD17B7P2	hydroxysteroid (17-beta) dehydrogenase 7 pseudogene 2	3.74	2.83	NM_002395.3	ME1	malic enzyme 1	2.29	2.31
NM_001013680.1	LOC401233	similar to HIV TAT specific factor 1	3.74	2.10	NM_001360.2	DHCR7	7-dehydrocholesterol reductase	2.29	2.73
NM_006918.4	SC5DL	sterol-C5-desaturase	3.68	3.41	NM_032421.2	CLIP2	CAP-GLY domain containing linker protein 2	2.27	1.79
NM_182980.2	OSGIN1	oxidative stress induced growth inhibitor 1	3.55	4.48	NM_014039.2	C11orf54	chromosome 11 open reading frame 54	2.27	1.62
NM_025182.2	KIAA1539	KIAA1539	3.49	2.62	NM_017585.2	SLC2A6	solute carrier family 2	2.27	2.35
NM_018677.2	ACSS2	acyl-CoA synthetase short-chain family member 2	3.46	4.68	NM_014330.2	PPP1R15A	protein phosphatase 1	2.26	1.63
NM_001034850.1	FAM134B	family with sequence similarity 134	3.44	1.90	NM_145306.2	C10orf35	chromosome 10 open reading frame 35	2.26	2.19
NM_153742.3	CTH	cystathionase (cystathionine gamma-lyase)	3.38	2.13	NM_003831.3	RIOK3	RIO kinase 3	2.25	1.93
NM_021158.3	TRIB3	tribbles homolog 3	3.34	3.71	NM_006997.2	TACC2	transforming, acidic coiled-coil containing protein 2	2.25	1.95
NM_000434.2	NEU1	sialidase 1	3.28	2.00	NM_001006932.1	RPS6KA2	ribosomal protein S6 kinase	2.24	1.68
NM_001005404.3	YPEL2	yippee-like 2	3.22	2.40	NM_000574.2	CD55	CD55 molecule	2.23	1.84
NM_004508.2	IDI1	isopentenyl-diphosphate delta isomerase 1	3.21	4.78	NM_018310.2	BRF2	subunit of RNA polymerase III transcription initiation factor	2.23	2.14
NM_003151.2	STAT4	signal transducer and activator of transcription 4	3.18	3.85	NM_001491.2	GCNT2	glucosaminyl (N-acetyl) transferase 2	2.22	4.08
NM_005165.2	ALDOC	aldolase C	3.16	2.44	NM_003567.2	BCAR3	breast cancer anti-estrogen resistance 3	2.21	1.79
NM_014331.3	SLC7A11	solute carrier family 7	3.16	2.54	NM_005488.1	TOM1	target of myb1	2.20	1.89
NM_000104.2	CYP1B1	cytochrome P450	3.15	2.41	NM_004462.3	FDFT1	farnesyl-diphosphate farnesyltransferase 1	2.19	2.73
NM_002340.3	LSS	lanosterol synthase	3.14	3.15	NM_019116.2	UBFD1	ubiquitin family domain containing 1	2.19	1.90
NM_078487.2	CDKN2B	cyclin-dependent kinase inhibitor 2B	3.13	2.13	NM_007105.1	SLC22A18AS	solute carrier family 22	2.18	1.81
NM_002201.4	ISG20	interferon stimulated exonuclease gene 20kDa	3.12	1.91	NM_182491.1	ZFAND2A	zinc finger	2.17	2.17
NM_016315.2	GULP1	GULP, engulfment adaptor PTB domain containing 1	3.11	4.39	NM_181785.2	SLC46A3	solute carrier family 46	2.17	2.00
NM_017983.4	WIPI1	WD repeat domain	3.09	2.40	NM_024866.4	ADM2	adrenomedullin 2	2.16	1.77
NM_024165.1	PHF1	PHD finger protein 1	3.07	2.42	NM_021133.2	RNASEL	ribonuclease L	2.15	2.28
NM_001995.2	ACSL1	acyl-CoA synthetase long-chain family member 1	3.06	2.86	NM_032548.2	ABTB1	ankyrin repeat and BTB (POZ) domain containing 1	2.14	1.58
NM_004354.1	CCNG2	cyclin G2	3.05	2.64	NM_002153.1	HSD17B2	hydroxysteroid (17-beta) dehydrogenase 2	2.14	2.98
NM_020739.2	CCPG1	cell cycle progression 1	3.04	1.88	NM_006033.2	LIPG	lipase	2.13	2.86
NM_001017369.1	SC4MOL	sterol-C4-methyl oxidase-like	3.03	4.04	NM_020919.2	ALS2	amyotrophic lateral sclerosis 2	2.11	1.77
NM_001031744.1	LOC158160	hypothetical protein LOC158160	3.02	2.35	NM_014701.2	KIAA0256	KIAA0256 gene product	2.11	2.30
NM_032409.2	PINK1	PTEN induced putative kinase 1	2.98	2.44	NM_001222.2	CAMK2G	calcium/calmodulin-dependent protein kinase	2.11	2.12
NM_003811.2	TNFSF9	tumor necrosis factor (ligand) superfamily	2.98	3.04	NM_015271.2	TRIM2	tripartite motif-containing 2	2.11	1.79
NM_002357.2	MXD1	MAX dimerization protein 1	2.93	2.30	NM_174936.2	PCSK9	proprotein convertase subtilisin/kexin type 9	2.11	4.18
NM_000777.2	CYP3A5	cytochrome P450	2.92	1.77	NM_015713.3	RRM2B	ribonucleotide reductase M2 B	2.10	1.66
NM_001098500.1	KIAA1217	KIAA1217	2.88	3.16	NM_005749.2	TOB1	transducer of ERBB2	2.09	1.58
NM_022119.3	PRSS22	protease	2.84	3.27	NM_004419.3	DUSP5	dual specificity phosphatase 5	2.08	3.61
NM_004669.2	CLIC3	Homo sapiens chloride intracellular channel 3	2.80	6.19	NM_033028.2	BBS4	Bardet-Biedl syndrome 4	2.08	2.04
NM_080491.1	GAB2	GRB2-associated binding protein 2	2.79	2.06	NM_001286.2	CLCN6	chloride channel 6	2.07	2.18
NM_178270.1	ATG4A	ATG4 autophagy related 4 homolog A	2.79	1.92	XM_001132495.1	SLC26A11	PREDICTED:solute carrier family 26	2.07	1.87
NM_002770.2	PRSS2	protease, serine, 2 (trypsin 2)	2.79	1.64	NM_172070.2	ZNF650	zinc finger protein 650	2.07	1.90
NM_182608.2	ANKRD33	ankyrin repeat domain 33	2.74	1.91	NM_004567.2	PFKFB4	6-phosphofructo-2-kinase/fructose-2	2.07	1.93
NM_006705.2	GADD45G	growth arrest and DNA-damage-inducible	2.74	3.01	NM_002555.3	SLC22A18	solute carrier family 22	2.06	1.62
NM_012326.2	MAPRE3	microtubule-associated protein	2.73	2.16	NM_001010990.1	HERPUD1	homocysteine-inducible, ubiquitin-like domain member 1	2.06	1.63
NM_001013251.1	SLC3A2	solute carrier family 3	2.72	2.40	NM_198867.1	ALKBH6	alkB, alkylation repair homolog 6	2.06	1.65
NM_016243.2	CYB5R1	cytochrome b5 reductase 1	2.72	2.52	NM_000271.3	NPC1	Niemann-Pick disease	2.05	2.18
NM_198129.1	LAMA3	laminin	2.65	3.36	NM_006454.2	MXD4	MAX dimerization protein 4	2.05	1.80
NM_000228.2	LAMB3	laminin	2.65	2.27	NM_021194.2	SLC30A1	solute carrier family 30	2.03	1.73
NM_006608.1	PHTF1	putative homeodomain transcription factor 1	2.64	2.38	NM_173843.1	IL1RN	interleukin 1 receptor antagonist	2.03	10.01
NM_021229.3	NTN4	netrin 4	2.03	2.47	NM_152271.3	LONRF1	LON peptidase N-terminal domain and ring finger 1	1.72	3.04
NM_002064.1	GLRX	glutaredoxin	2.03	1.75	NM_003314.1	TTC1	tetratricopeptide repeat domain 1	1.72	1.55
NM_005896.2	IDH1	isocitrate dehydrogenase 1	2.02	2.37	NM_005920.2	MEF2D	myocyte enhancer factor 2D	1.71	1.85
NM_144498.1	OSBPL2	oxysterol binding protein-like 2	2.01	1.81	NM_016410.2	CHMP5	chromatin modifying protein 5	1.71	2.14
NM_005115.3	MVP	major vault protein	1.99	1.82	NM_001748.3	CAPN2	calpain 2	1.70	2.16
NM_023938.4	C1orf116	chromosome 1 open reading frame 116	1.98	3.06	NM_004344.1	CETN2	centrin	1.70	1.54
NM_024311.2	MFSD11	major facilitator superfamily domain containing 11	1.98	1.80	NM_177947.2	ARMCX3	armadillo repeat containing, X-linked 3	1.70	1.76
NM_004433.3	ELF3	E74-like factor 3	1.97	3.72	NM_000158.2	GBE1	glucan	1.69	1.73
NM_031229.2	RBCK1	RanBP-type and C3HC4-type zinc finger containing 1	1.97	4.21	NM_052873.1	C14orf179	chromosome 14 open reading frame 179	1.69	1.52
NM_005564.3	LCN2	lipocalin 2	1.97	4.19	NM_020310.2	MNT	MAX binding protein	1.69	1.51
NM_002956.2	CLIP1	CAP-GLY domain containing linker protein 1	1.96	1.62	NM_015367.2	BCL2L13	BCL2-like 13	1.69	1.88
NM_005562.1	LAMC2	laminin, gamma 2	1.96	2.01	NM_001005474.1	NFKBIZ	nuclear factor of kappa light polypeptide gene enhancer	1.69	1.72
NM_000332.2	ATXN1	ataxin 1	1.96	1.86	NM_021202.1	TP53INP2	tumor protein p53 inducible nuclear protein 2	1.69	1.89
NM_001206.2	KLF9	Kruppel-like factor 9	1.96	2.19	NM_138448.2	ACYP2	acylphosphatase 2	1.68	2.03
NM_173359.3	EIF4E3	eukaryotic translation initiation factor 4E family member 3	1.95	2.01	NM_003729.1	RTCD1	RNA terminal phosphate cyclase domain 1	1.67	1.61
NM_003565.1	ULK1	unc-51-like kinase 1	1.95	1.57	NM_030912.2	TRIM8	tripartite motif-containing 8	1.67	1.70
NM_012161.2	FBXL5	F-box and leucine-rich repeat protein 5	1.95	2.27	NM_018202.3	TMEM57	transmembrane protein 57	1.67	1.88
NM_001079864.1	TAX1BP1	Tax1 (human T-cell leukemia virus type I) binding protein 1	1.93	1.66	NM_018297.2	NGLY1	N-glycanase 1	1.66	1.68
NM_005063.4	SCD	stearoyl-CoA desaturase	1.93	4.44	NM_183399.1	RNF14	ring finger protein 14	1.66	1.87
NM_145279.4	MOBKL2C	MOB1, Mps One Binder kinase activator-like 2C	1.92	2.58	NM_032357.2	CCDC115	coiled-coil domain containing 115	1.66	1.63
NM_004055.4	CAPN5	calpain 5	1.91	2.05	NM_172037.2	RDH10	retinol dehydrogenase 10	1.66	2.20
NM_175932.1	PSMD13	proteasome (prosome, macropain) 26S subunit	1.91	2.14	NM_012287.3	CENTB2	centaurin	1.65	1.62
NM_002885.1	RAP1GAP	RAP1 GTPase activating protein	1.91	2.02	NM_018178.3	GOLPH3L	golgi phosphoprotein 3-like	1.65	1.64
NM_145245.2	EVI5L	ecotropic viral integration site 5-like	1.90	1.69	NM_025147.3	COQ10B	coenzyme Q10 homolog B	1.64	1.54
NM_021242.3	MID1IP1	MID1 interacting protein 1	1.89	1.78	NM_006285.2	TESK1	testis-specific kinase 1	1.64	1.61
NM_006503.2	PSMC4	proteasome (prosome, macropain) 26S subunit	1.89	2.16	NM_001018102.1	GRINL1A	glutamate receptor	1.64	1.92
NM_005975.2	PTK6	protein tyrosine kinase 6	1.88	1.97	NM_005476.3	GNE	glucosamine (UDP-N-acetyl)-2-epimerase	1.64	2.98
NM_207304.1	MBNL2	muscleblind-like 2	1.88	1.63	NR_002204.1	FTHL11	ferritin, heavy polypeptide-like 11 on chromosome 8	1.63	1.79
NM_000859.1	HMGCR	3-hydroxy-3-methylglutaryl-Coenzyme A reductase	1.88	2.46	NM_053067.1	UBQLN1	ubiquilin 1	1.63	1.56
NM_021021.2	SNTB1	syntrophin	1.88	1.64	NM_005895.3	GOLGA3	golgi autoantigen	1.63	1.58
NM_032667.4	BSCL2	Bernardinelli-Seip congenital lipodystrophy 2	1.87	1.73	NM_037370.1	CCNDBP1	cyclin D-type binding-protein 1	1.63	1.50
NM_006022.2	TSC22D1	TSC22 domain family	1.87	2.40	NM_001042430.1	FAM164C	family with sequence similarity 164	1.62	2.01
NM_012478.3	WBP2	WW domain binding protein 2	1.87	1.67	NM_004751.1	GCNT3	glucosaminyl (N-acetyl) transferase 3	1.62	3.99
NM_031476.2	CRISPLD2	cysteine-rich secretory protein LCCL domain containing 2	1.87	2.57	NM_004428.2	EFNA1	ephrin-A1	1.61	2.10
NM_017921.1	NPLOC4	nuclear protein localization 4 homolog	1.87	1.74	NM_007193.3	ANXA10	annexin A10	1.61	2.36
NM_015508.3	TIPARP	TCDD-inducible poly(ADP-ribose) polymerase	1.86	1.75	NM_018180.2	DHX32	DEAH (Asp-Glu-Ala-His) box polypeptide 32	1.61	1.52
NM_001376.2	DYNC1H1	dynein, cytoplasmic 1, heavy chain 1	1.86	1.50	NM_020202.2	NIT2	nitrilase family	1.61	1.62
NM_144693.1	ZNF558	zinc finger protein 558	1.86	1.98	NM_032169.4	ACAD11	acyl-Coenzyme A dehydrogenase family	1.61	1.73
NM_003344.2	UBE2H	ubiquitin-conjugating enzyme E2H	1.85	2.04	NM_147223.2	NCOA1	nuclear receptor coactivator 1	1.60	1.57
NM_152528.1	WDSUB1	WD repeat, sterile alpha motif and U-box domain containing 1	1.85	1.74	NM_002203.3	ITGA2	integrin	1.59	2.49
NM_001225.3	CASP4	caspase 4, apoptosis-related cysteine peptidase	1.85	1.97	NM_018317.1	TBC1D19	TBC1 domain family	1.59	2.18
NM_001512.2	GSTA4	glutathione S-transferase A4	1.84	2.45	NM_002061.2	GCLM	glutamate-cysteine ligase	1.59	2.06
NM_020299.3	AKR1B10	aldo-keto reductase family 1	1.83	2.42	NM_019007.3	ARMCX6	armadillo repeat containing, X-linked 6	1.58	1.81
NM_016143.3	NSFL1C	NSFL1 (p97) cofactor (p47)	1.83	2.61	NM_015974.2	CRYL1	crystallin, lambda 1	1.58	1.51
NM_080655.1	C9orf30	chromosome 9 open reading frame 30	1.83	1.77	NM_006149.2	LGALS4	lectin	1.58	3.12
NM_001018109.1	PIR	pirin	1.83	2.67	NM_012233.1	RAB3GAP1	RAB3 GTPase activating protein subunit 1	1.57	1.78
NR_002200.1	FTHL2	ferritin, heavy polypeptide-like 2 on chromosome 1	1.83	1.62	NM_002808.3	PSMD2	proteasome (prosome, macropain) 26S subunit	1.57	1.72
NM_005485.3	PARP3	poly (ADP-ribose) polymerase family	1.82	1.77	NM_001030001.1	RPS29	ribosomal protein S29	1.57	1.61
NM_058172.3	ANTXR2	anthrax toxin receptor 2	1.82	1.88	NM_052849.2	CCDC32	coiled-coil domain containing 32	1.57	1.55
NR_002166.1	SEDLP	spondyloepiphyseal dysplasia, late, pseudogene	1.81	1.88	NM_015484.4	SYF2	SYF2 homolog	1.57	1.64
NM_022449.1	RAB17	member RAS oncogene family	1.80	1.64	NM_001093771.1	TXNRD1	thioredoxin reductase 1	1.57	1.54
NM_198310.2	TTC8	tetratricopeptide repeat domain 8	1.80	1.83	NM_017707.2	ASAP3	ArfGAP with SH3 domain, ankyrin repeat and PH domain 3	1.57	1.59
NM_002786.2	PSMA1	proteasome (prosome, macropain) subunit	1.79	2.54	NM_020412.3	CHMP1B	chromatin modifying protein 1B	1.56	1.83
NM_002796.2	PSMB4	proteasome (prosome, macropain) subunit	1.78	1.53	NM_017549.3	EPDR1	ependymin related protein 1	1.56	1.52
NR_002201.1	FTHL3P	ferritin	1.78	2.10	NM_001080493.2	ZNF823	zinc finger protein 823	1.56	1.56
NM_020225.1	STOX2	storkhead box 2	1.78	5.98	NM_001008738.2	FNIP1	folliculin interacting protein 1	1.56	1.50
NM_080725.1	SRXN1	sulfiredoxin 1 homolog	1.78	1.67	NM_006343.2	MERTK	c-mer proto-oncogene tyrosine kinase	1.56	2.12
NM_015946.4	PELO	pelota homolog	1.77	1.58	NM_014851.2	KLHL21	kelch-like 21	1.56	2.02
NM_020751.1	COG6	component of oligomeric golgi complex 6	1.77	1.60	NM_002803.2	PSMC2	proteasome	1.55	1.77
NM_001080538.1	LOC441282	similar to aldo-keto reductase family 1	1.77	2.31	NM_145918.2	CTSL1	cathepsin L1	1.55	1.96
NM_004487.3	GOLGB1	golgin B1	1.76	1.82	NM_003846.1	PEX11B	peroxisomal biogenesis factor 11 beta	1.55	1.92
NM_145040.2	PRKCDBP	protein kinase C	1.76	3.27	NM_014905.2	GLS	glutaminase	1.55	2.21
NM_201557.2	FHL2	four and a half LIM domains 2	1.76	1.74	NM_016154.3	RAB4B	member RAS oncogene family	1.55	2.22
NM_015037.2	KIAA0913	KIAA0913	1.75	1.52	NM_052901.2	SLC25A25	solute carrier family 25	1.54	1.55
NM_003003.2	SEC14L1	SEC14-like 1	1.74	1.94	NM_033212.2	CCDC102A	coiled-coil domain containing 102A	1.54	1.75
NM_001031835.1	PHKB	phosphorylase kinase	1.74	1.63	NM_002799.2	PSMB7	proteasome (prosome, macropain) subunit	1.54	1.56
NM_016470.6	C20orf111	chromosome 20 open reading frame 111	1.74	1.54	NM_001031716.1	OBFC2A	oligonucleotide/oligosaccharide-binding fold containing 2A	1.53	1.60
NM_001080791.1	C15orf57	chromosome 15 open reading frame 57	1.74	1.63	NM_015187.3	KIAA0746	KIAA0746 protein	1.53	1.62
NM_002541.2	OGDH	oxoglutarate (alpha-ketoglutarate) dehydrogenase	1.74	1.55	NM_004034.1	ANXA7	annexin A7	1.52	1.75
NM_015922.1	NSDHL	NAD(P) dependent steroid dehydrogenase-like	1.74	2.30	NM_002815.2	PSMD11	proteasome (prosome, macropain) 26S subunit	1.52	1.71
XM_001128220.1	PLEKHM1	pleckstrin homology domain containing, family M member 1	1.73	1.73	NM_014889.2	PITRM1	pitrilysin metallopeptidase 1	1.52	2.15
NM_004417.2	DUSP1	dual specificity phosphatase 1	1.73	1.78	NM_007126.2	VCP	valosin-containing protein	1.51	1.79
NM_003971.3	SPAG9	sperm associated antigen 9	1.73	1.80	NM_032017.1	STK40	serine/threonine kinase 40	1.50	1.66
NM_016026.2	RDH11	retinol dehydrogenase 11	1.72	1.67	NM_032776.1	JMJD1C	jumonji domain containing 1C	1.50	1.81

**Table 2:** Down-regulated genes (p<0.001) common in GANT61-treated HT29 and GC3/c1 cells.

ACCESSION NUMBER	GENE SYMBOL	DEFINITION	Fold Change		ACCESSION NUMBER	GENE SYMBOL	DEFINITION	Fold Change	
			HT29	GC3/c1				HT29	GC3/c1
NM_015086.1	DDN	dendrin	−6.81	−2.37	NM_014791.2	MELK	maternal embryonic leucine zipper kinase	−2.50	−2.09
NM_001013653.2	LRRC26	leucine rich repeat containing 26	−4.77	−2.23	NM_016343.3	CENPF	centromere protein F, 350/400ka	−2.50	−2.71
NM_004091.2	E2F2	E2F transcription factor 2	−4.23	−2.26	NM_001789.2	CDC25A	cell division cycle 25 homolog A	−2.50	−2.45
NM_182687.1	PKMYT1	protein kinase, membrane associated tyrosine/threonine 1	−4.17	−4.81	NM_024037.1	C1orf135	chromosome 1 open reading frame 135	−2.49	−2.12
NM_001013653.1	LOC389816	cytokeratin associated protein	−3.85	−2.32	NM_017669.2	ERCC6L	excision repair cross-complementing rodent repair deficiency	−2.48	−3.22
NM_020675.3	SPC25	NDC80 kinetochore complex component, homolog	−3.51	−7.19	NM_031965.2	GSG2	germ cell associated 2 (haspin)	-2.47	−2.79
NM_005733.1	KIF20A	kinesin family member 20A	−3.45	−2.21	NM_018186.2	C1orf112	chromosome 1 open reading frame 112	−2.47	−1.88
NM_002263.2	KIFC1	kinesin family member C1	−3.36	−2.74	NM_001786.2	CDC2	cell division cycle 2, G1 to S and G2 to M	−2.47	−2.63
NM_006681.1	NMU	neuromedin U	−3.33	−3.15	NM_002105.2	H2AFX	H2A histone family, member X	−2.46	−2.86
NM_014783.2	ARHGAP11A	Rho GTPase activating protein 11A	−3.33	−3.46	NM_018492.2	PBK	PDZ binding kinase	−2.46	−2.21
NM_006176.1	NRGN	neurogranin	−3.29	−4.20	NM_198516.1	GALNTL4	UDP-N-acetyl-alpha-D-galactosamine	−2.46	−1.82
NM_016095.1	GINS2	GINS complex subunit 2	−3.25	−4.51	NM_018685.2	ANLN	anillin, actin binding protein	−2.46	−2.44
NM_018063.3	HELLS	helicase, lymphoid-specific	−3.24	−3.42	NM_002915.3	RFC3	replication factor C (activator 1) 3, 38kDa	−2.45	−2.59
NM_057735.1	CCNE2	cyclin E2	−3.22	−3.24	NM_007370.3	RFC5	replication factor C (activator 1) 5, 36.5kDa	−2.43	−2.28
NM_014750.3	DLG7	discs, large homolog 7	−3.21	−2.48	NM_001032290.1	PSRC1	proline/serine-rich coiled-coil 1	−2.43	−2.53
NM_001168.2	BIRC5	baculoviral IAP repeat-containing 5	−3.18	−2.79	NM_000057.2	BLM	Bloom syndrome	−2.43	−3.28
NM_001255.2	CDC20	cell division cycle 20 homolog	−3.12	−2.46	NM_012484.1	HMMR	hyaluronan-mediated motility receptor	−2.43	−1.82
NM_001237.2	CCNA2	cyclin A2	−3.11	−3.01	NM_145701.1	CDCA4	cell division cycle associated 4	−2.43	−2.26
NM_144508.3	CASC5	cancer susceptibility candidate 5	−3.09	−2.13	NM_024339.2	THOC6	THO complex 6 homolog	−2.41	−1.74
NM_017611.2	SLC43A3	solute carrier family 43, member 3	−3.05	−2.60	NM_003258.2	TK1	thymidine kinase 1	−2.41	−3.74
NM_018154.2	ASF1B	ASF1 anti-silencing function 1 homolog B	−3.03	−3.06	NM_182776.1	MCM7	minichromosome maintenance complex component 7	−2.39	−2.54
NM_018410.3	HJURP	Holliday junction recognition protein	−3.02	−3.18	NM_178448.2	C9orf140	chromosome 9 open reading frame 140	−2.39	−2.34
NM_004217.2	AURKB	aurora kinase B	−3.01	−3.19	NM_001067.2	TOP2A	topoisomerase (DNA) II alpha 170kDa	−2.39	−3.16
NM_002692.2	POLE2	polymerase (DNA directed), epsilon 2 (p59 subunit)	−3.01	−3.00	NM_178014.2	TUBB	tubulin, beta	−2.37	−2.27
NM_018101.2	CDCA8	cell division cycle associated 8	−2.97	−2.85	NM_018193.2	FANCI	Fanconi anemia, complementation group I	−2.37	−2.52
NM_002875.2	RAD51	RAD51 homolog	−2.95	−2.66	NM_206833.2	CTXN1	cortexin 1	−2.36	−2.04
NM_031299.3	CDCA3	cell division cycle associated 3	−2.94	−2.82	NM_031966.2	CCNB1	cyclin B1	−2.36	−2.28
NM_181803.1	UBE2C	ubiquitin-conjugating enzyme E2C	−2.91	−2.80	NM_005483.2	CHAF1A	chromatin assembly factor 1, subunit A (p150)	−2.36	−2.21
NM_001100118.1	XRCC3	X-ray repair complementing defective repair 3	−2.90	−3.66	NM_005518.2	HMGCS2	3-hydroxy-3-methylglutaryl-Coenzyme A synthase 2	−2.35	−2.88
NM_025049.2	PIF1	PIF1 5′-to-3′ DNA helicase homolog	−2.90	−2.87	NM_019013.1	FAM64A	family with sequence similarity 64	−2.35	−3.13
NM_030928.2	CDT1	chromatin licensing and DNA replication factor 1	−2.86	−2.76	NM_002129.2	HMGB2	high-mobility group box 2	−2.35	−2.18
NM_016556.1	PSMC3IP	PSMC3 interacting protein	−2.85	−3.47	NM_001034194.1	EXOSC9	exosome component 9	−2.34	−1.69
NM_182746.1	MCM4	minichromosome maintenance complex component 4	−2.84	−2.97	NM_031423.3	NUF2	NDC80 kinetochore complex component, homolog	−2.34	−2.48
NM_016937.2	POLA1	polymerase (DNA directed), alpha 1, catalytic subunit	−2.84	−2.19	NM_002916.3	RFC4	replication factor C (activator 1) 4, 37kDa	−2.33	−2.36
NM_007086.2	WDHD1	WD repeat and HMG-box DNA binding protein 1	−2.80	−2.53	NM_004111.4	FEN1	flap structure-specific endonuclease 1	−2.33	−1.97
NM_001761.1	CCNF	cyclin F	−2.80	−2.77	NM_145061.3	C13orf3	chromosome 13 open reading frame 3	−2.33	−2.53
NM_012177.2	FBXO5	F-box protein 5	−2.78	−2.95	NM_001012413.1	SGOL1	shugoshin-like 1	−2.33	−2.85
NM_018518.3	MCM10	minichromosome maintenance complex component 10	−2.78	−4.01	NM_207418.2	GCUD2	gastric cancer up-regulated-2	−2.32	−2.28
NM_003579.2	RAD54L	RAD54-like	−2.76	−2.98	NM_199413.1	PRC1	protein regulator of cytokinesis 1	−2.32	−2.13
NM_020242.1	KIF15	kinesin family member 15	−2.75	−3.00	NM_006461.3	SPAG5	sperm associated antigen 5	−2.31	−1.92
NM_004336.2	BUB1	BUB1 budding uninhibited by benzimidazoles 1 homolog	−2.74	−2.47	NM_080668.2	CDCA5	cell division cycle associated 5	−2.29	−2.94
NM_003384.2	VRK1	vaccinia related kinase 1	−2.73	−1.89	NM_006101.1	NDC80	NDC80 homolog	−2.29	−3.12
NM_018454.5	NUSAP1	nucleolar and spindle associated protein 1	−2.72	−2.40	NM_001012507.1	C6orf173	chromosome 6 open reading frame 173	−2.29	−2.38
NM_001034.1	RRM2	ribonucleotide reductase M2 polypeptide	−2.72	−2.54	NM_002497.2	NEK2	NIMA (never in mitosis gene a)-related kinase 2	−2.29	−2.10
NM_012112.4	TPX2	microtubule-associated, homolog	−2.72	−2.32	NM_024900.3	PHF17	PHD finger protein 17	−2.28	−1.67
NM_152259.3	C15orf42	chromosome 15 open reading frame 42	−2.70	−3.44	NM_001040668.1	BCL2L12	BCL2-like 12	−2.28	−1.93
NM_001042426.1	CENPA	centromere protein A	−2.70	−3.28	NM_014264.3	PLK4	polo-like kinase 4	−2.27	−1.97
NM_012415.2	RAD54B	RAD54 homolog B	−2.69	−1.88	NM_022809.2	CDC25C	cell division cycle 25 homolog C	−2.27	−2.05
NM_181471.1	RFC2	replication factor C (activator 1) 2, 40kDa	−2.69	−2.50	NM_152515.2	CKAP2L	cytoskeleton associated protein 2-like	−2.26	−2.29
NM_005480.2	TROAP	trophinin associated protein	−2.69	−2.49	NM_006026.2	H1FX	H1 histone family, member X	−2.24	−2.08
NM_006845.2	KIF2C	kinesin family member 2C	−2.68	−2.81	NM_003276.1	TMPO	thymopoietin	−2.24	−1.95
NM_022346.3	NCAPG	non-SMC condensin I complex	−2.68	−2.70	NM_018136.3	ASPM	asp (abnormal spindle) homolog	−2.23	−2.51
NM_004260.2	RECQL4	RecQ protein-like 4	−2.68	−3.00	NM_016426.4	GTSE1	G-2 and S-phase expressed 1	−2.23	−2.44
NM_003318.3	TTK	TTK protein kinase	−2.67	−2.40	NM_014875.1	KIF14	kinesin family member 14	−2.23	−2.12
NM_030919.2	FAM83D	family with sequence similarity 83, member D	−2.66	−2.08	NM_014109.2	ATAD2	ATPase family, AAA domain containing 2	−2.23	−2.21
NM_001254.3	CDC6	cell division cycle 6 homolog	−2.66	−3.05	NM_003390.2	WEE1	WEE1 homolog	−2.22	−1.97
NM_001034836.1	RDM1	RAD52 motif 1	−2.65	−2.65	NM_002388.3	MCM3	minichromosome maintenance complex component 3	−2.22	−2.45
NM_004526.2	MCM2	minichromosome maintenance complex component 2	−2.65	−3.43	NM_005030.3	PLK1	polo-like kinase 1	−2.22	−2.37
NM_004701.2	CCNB2	cyclin B2	−2.65	−2.52	NM_005375.2	MYB	v-myb myeloblastosis viral oncogene homolog	−2.22	−2.01
NM_032818.2	C9orf100	chromosome 9 open reading frame 100	−2.64	−2.30	NM_024789.3	TMEM180	transmembrane protein 180	−2.20	−1.63
NM_006397.2	RNASEH2A	ribonuclease H2, subunit A	−2.64	−2.52	NM_001012716.1	C18orf56	chromosome 18 open reading frame 56	−2.20	−2.30
NM_014736.4	KIAA0101	KIAA0101	−2.61	−3.47	NM_017760.5	NCAPG2	non-SMC condensin II complex, subunit G2	−2.20	−2.11
NM_004219.2	PTTG1	pituitary tumor-transforming 1	−2.61	−2.23	NM_005192.2	CDKN3	cyclin-dependent kinase inhibitor 3	−2.20	−1.98
NM_182513.1	SPC24	SPC24, NDC80 kinetochore complex component, homolog	−2.61	−3.15	NM_006231.2	POLE	polymerase (DNA directed)	−2.19	−3.53
NM_198948.1	NUDT1	nudix (nucleoside diphosphate linked moiety X)-type motif 1	−2.59	−3.09	NM_018124.3	RFWD3	ring finger and WD repeat domain 3	−2.19	−1.56
NM_033286.2	C15orf23	chromosome 15 open reading frame 23	−2.58	−1.77	NM_006088.5	TUBB2C	tubulin, beta 2C	−2.19	−1.63
NM_024053.3	CENPM	centromere protein M	−2.57	−3.45	NM_198434.1	AURKA	aurora kinase A	−2.18	−1.68
NM_006479.3	RAD51AP1	RAD51 associated protein 1	−2.57	−2.54	NM_007280.1	OIP5	Opa interacting protein 5	−2.18	−2.34
NM_006739.3	MCM5	minichromosome maintenance complex component 5	−2.56	−3.21	NM_014176.2	UBE2T	ubiquitin-conjugating enzyme E2T (putative).	−2.15	−2.01
NM_203401.1	STMN1	stathmin 1/oncoprotein 18	−2.56	−2.32	NM_199420.3	POLQ	polymerase (DNA directed), theta	−2.14	−2.45
NM_013277.2	RACGAP1	Rac GTPase activating protein 1	−2.55	−1.79	NM_001211.4	BUB1B	BUB1 budding uninhibited by benzimidazoles 1 homolog beta	−2.13	−2.23
NM_001005413.1	ZWINT	ZW10 interactor	−2.55	−2.64	NM_001025248.1	DUT	deoxyuridine triphosphatase	−2.13	−2.36
NM_004856.4	KIF23	kinesin family member 23	−2.54	−2.17	NM_031217.2	KIF18A	kinesin family member 18A	−2.13	−1.99
NM_004523.2	KIF11	kinesin family member 11	−2.53	−2.12	NM_025108.2	C16orf59	chromosome 16 open reading frame 59	−2.12	−3.48
NR_002734.1	PTTG3	pituitary tumor-transforming 3	−2.53	−2.43	NM_001042517.1	DIAPH3	diaphanous homolog 3	−2.12	−2.34
NM_006027.3	EXO1	exonuclease 1	−2.52	−3.86	NM_017998.1	C9orf40	chromosome 9 open reading frame 40	−2.11	−1.85
NM_016448.1	DTL	denticleless homolog	−2.51	−3.12	NM_052844.3	WDR34	WD repeat domain 34	−2.11	−2.71
NM_022770.2	GINS3	GINS complex subunit 3 (Psf3 homolog)	−2.10	−1.65	NM_017918.3	CCDC109B	coiled-coil domain containing 109B	−1.84	−1.84
NM_003503.2	CDC7	cell division cycle 7 homolog	−2.09	−1.97	NM_005782.2	THOC4	THO complex 4	−1.84	−1.63
NM_001071.1	TYMS	thymidylate synthetase	−2.08	−3.41	NM_003035.2	STIL	SCL/TAL1 interrupting locus	−1.84	−1.95
NM_005441.2	CHAF1B	chromatin assembly factor 1, subunit B (p60)	−2.08	−2.25	NM_005342.2	HMGB3	high-mobility group box 3	−1.83	−1.60
NM_014865.2	NCAPD2	non-SMC condensin I complex, subunit D2	−2.07	−2.03	NM_018132.3	CENPQ	centromere protein Q	−1.83	−2.10
NM_001798.2	CDK2	cyclin-dependent kinase 2	−2.07	−1.88	NM_024516.2	C16orf53	chromosome 16 open reading frame 53	−1.81	−1.86
NM_022720.5	DGCR8	DiGeorge syndrome critical region gene 8	−2.07	−1.57	NM_015895.3	GMNN	geminin, DNA replication inhibitor	−1.81	−1.87
NM_145018.2	C11orf82	chromosome 11 open reading frame 82	−2.06	−2.28	NM_002689.2	POLA2	polymerase (DNA directed), alpha 2 (70kD subunit)	−1.81	−2.11
NM_016195.2	KIF20B	kinesin family member 20B	−2.06	−1.63	NM_000154.1	GALK1	galactokinase 1	−1.80	−1.84
NM_001008393.1	LOC201725	hypothetical protein	−2.05	−1.98	NM_004499.3	HNRNPAB	heterogeneous nuclear ribonucleoprotein A/B	−1.80	−2.31
NM_000465.1	BARD1	BRCA1 associated RING domain 1	−2.05	−2.42	NM_024656.2	GLT25D1	glycosyltransferase 25 domain containing 1	−1.80	−1.62
NM_003173.2	SUV39H1	suppressor of variegation 3-9 homolog 1	−2.05	−1.89	NM_199250.1	C19orf48	chromosome 19 open reading frame 48	−1.79	−1.83
NM_015426.2	WDR51A	WD repeat domain 51A	−2.05	−1.60	NM_153329.2	ALDH16A1	aldehyde dehydrogenase 16 family, member A1	−1.79	−1.98
NM_020342.1	SLC39A10	solute carrier family 39	−2.05	−3.15	NM_032637.2	SKP2	S-phase kinase-associated protein 2 (p45)	−1.79	−2.00
NM_144999.2	LRRC45	leucine rich repeat containing 45	−2.04	−3.33	NM_006191.2	PA2G4	proliferation-associated 2G4, 38kDa	−1.78	−1.84
NM_000946.2	PRIM1	primase, DNA, polypeptide 1 (49kDa)	−2.04	−2.29	NM_001033580.1	MYO19	myosin XIX	−1.78	−3.61
NM_016183.3	MRTO4	turnover 4 homolog	−2.04	−1.68	NM_017915.2	C12orf48	chromosome 12 open reading frame 48	−1.78	−1.92
NM_017518.5	UCHL5IP	UCHL5 interacting protein	−2.04	−1.75	NM_003362.2	UNG	uracil-DNA glycosylase	−1.76	−1.55
NM_020394.2	ZNF695	zinc finger protein 695	−2.03	−2.78	NM_014985.2	CEP152	centrosomal protein 152kDa	−1.75	−2.37
NM_203394.2	E2F7	E2F transcription factor 7	−2.02	−2.03	NM_001333.2	CTSL2	cathepsin L2	−1.75	−1.70
NM_173529.3	C18orf54	chromosome 18 open reading frame 54	−2.02	−1.99	NM_016310.2	POLR3K	polymerase (RNA) III (DNA directed) polypeptide K, 12.3 kDa	−1.75	−1.59
NM_000156.4	GAMT	guanidinoacetate N-methyltransferase	−2.02	−1.64	NM_006406.1	PRDX4	peroxiredoxin 4	−1.74	−1.93
NM_007299.2	BRCA1	breast cancer 1, early onset	−2.02	−2.11	NM_005484.2	PARP2	poly (ADP-ribose) polymerase family, member 2	−1.74	−1.53
NM_014641.1	MDC1	mediator of DNA damage checkpoint 1	−2.01	−1.57	NM_001039091.1	PRPS2	phosphoribosyl pyrophosphate synthetase 2	−1.73	−1.60
NM_032485.4	MCM8	minichromosome maintenance complex component 8	−2.01	−3.18	NM_015703.3	RRP7A	ribosomal RNA processing 7 homolog A	−1.73	−1.71
NM_024660.2	TMEM149	transmembrane protein 149	−2.01	−2.07	NM_015032.1	PDS5B	PDS5, regulator of cohesion maintenance, homolog B	−1.73	−1.78
NM_015414.2	RPL36	ribosomal protein L36	−2.00	−2.40	NM_000992.2	RPL29	ribosomal protein L29	−1.73	−2.53
NM_021734.3	SLC25A19	solute carrier family 25, member 19	−2.00	−1.67	NM_173608.1	C14orf80	chromosome 14 open reading frame 80	−1.72	−1.73
NM_003504.3	CDC45L	CDC45 cell division cycle 45-like	−2.00	−2.84	NM_001042550.1	SMC2	structural maintenance of chromosomes 2	−1.72	−1.92
NM_014708.3	KNTC1	kinetochore associated 1	−1.99	−1.86	NM_014285.4	EXOSC2	exosome component 2	−1.72	−1.70
NM_018131.3	CEP55	centrosomal protein 55kDa	−1.98	−2.01	NM_138961.1	ESAM	endothelial cell adhesion molecule	−1.71	−2.21
NM_007243.1	NRM	nurim	−1.98	−2.22	NM_145159.1	JAG2	jagged 2	−1.70	−1.89
NM_003517.2	HIST2H2AC	histone cluster 2, H2ac	−1.98	−2.54	NM_006392.2	NOL5A	nucleolar protein 5A (56kDa with KKE/D repeat)	−1.69	−1.70
NM_001002800.1	SMC4	structural maintenance of chromosomes 4	−1.98	−1.94	NM_033661.3	WDR4	WD repeat domain 4	−1.69	−1.84
NM_001033505.1	CSTF3	cleavage stimulation factor, 3′ pre-RNA, subunit 3, 77kD	−1.97	−1.96	NM_032358.2	CCDC77	coiled-coil domain containing 77	−1.68	−1.54
NM_181716.2	PRR6	proline rich 6	−1.96	−1.85	NM_018693.2	FBXO11	F-box protein 11	−1.68	−1.93
NM_018663.1	PXMP2	peroxisomal membrane protein 2, 22kDa	−1.96	−2.02	NM_001026.3	RPS24	ribosomal protein S24	−1.68	−1.94
NM_005915.4	MCM6	minichromosome maintenance complex component 6	−1.96	−2.25	NM_016292.1	TRAP1	TNF receptor-associated protein 1	−1.67	−1.51
NM_005189.1	CBX2	chromobox homolog 2	−1.96	−1.68	NM_017613.2	DONSON	downstream neighbor of SON	−1.66	−1.61
NM_001379.1	DNMT1	DNA (cytosine-5-)-methyltransferase 1	−1.96	−1.84	NM_006047.4	RBM12	RNA binding motif protein 12	−1.66	−1.91
NM_001002018.1	HCFC1R1	host cell factor C1 regulator 1 (XPO1 dependent)	−1.96	−1.79	NM_012140.3	SLC25A10	solute carrier family 25 (dicarboxylate transporter), member 10	−1.66	−1.55
NM_000234.1	LIG1	ligase I, DNA, ATP-dependent	−1.96	−2.81	NM_001025238.1	TSPAN4	tetraspanin 4	−1.66	−1.94
NM_138443.2	CCDC5	coiled-coil domain containing 5 (spindle associated)	−1.95	−1.68	NM_001008735.1	HMG1L1	high-mobility group (nonhistone chromosomal) protein 1-like 1	−1.66	−1.62
NM_020315.4	PDXP	pyridoxal (pyridoxine, vitamin B6) phosphatase	−1.95	−2.04	NM_005956.2	MTHFD1	methylenetetrahydrofolate dehydrogenase (NADP+dependent) 1	−1.65	−1.69
NM_032117.2	MND1	meiotic nuclear divisions 1 homolog	−1.95	−2.63	NM_145014.1	HYLS1	hydrolethalus syndrome 1	−1.65	−1.62
NM_194255.1	SLC19A1	solute carrier family 19 (folate transporter), member 1	−1.95	−1.93	NM_004309.3	ARHGDIA	Rho GDP dissociation inhibitor (GDI) alpha	−1.64	−1.75
NM_058216.1	RAD51C	RAD51 homolog C	−1.94	−2.13	NM_182649.1	PCNA	proliferating cell nuclear antigen	−1.63	−1.99
NM_018725.3	IL17RB	interleukin 17 receptor B	−1.94	−2.25	NM_032118.2	WDR54	WD repeat domain 54	−1.63	−1.77
NM_024844.3	NUP85	nucleoporin 85kDa	−1.93	−1.53	NM_015697.6	COQ2	coenzyme Q2 homolog	−1.63	−1.85
NM_015190.3	DNAJC9	DnaJ (Hsp40) homolog, subfamily C, member 9	−1.93	−1.64	NM_032982.2	CASP2	caspase 2	−1.63	−1.67
NM_004237.2	TRIP13	thyroid hormone receptor interactor 13	−1.93	−2.23	NM_001042762.1	FIGNL1	fidgetin-like 1	−1.63	−1.85
NM_000179.1	MSH6	mutS homolog 6	−1.93	−1.83	NM_001100417.1	UBR7	ubiquitin protein ligase E3 component n-recognin 7 (putative)	−1.61	−1.64
NM_001009936.1	PHF19	PHD finger protein 19	−1.93	−2.42	NM_003146.2	SSRP1	structure specific recognition protein 1	−1.60	−1.66
NM_003609.2	HIRIP3	HIRA interacting protein 3	−1.92	−2.01	NM_152308.1	C16orf75	chromosome 16 open reading frame 75	−1.60	−1.99
NM_022145.3	CENPK	centromere protein K	−1.92	−2.25	NM_001024662.1	RPL6	ribosomal protein L6	−1.60	−1.56
NM_022908.1	NT5DC2	5′-nucleotidase domain containing 2	−1.92	−1.81	NM_182620.3	FAM33A	family with sequence similarity 33, member A	−1.59	−2.17
NM_006342.1	TACC3	transforming, acidic coiled-coil containing protein 3	−1.92	−2.27	NM_033120.2	NKD2	naked cuticle homolog 2	−1.59	−1.65
NM_001080450.1	KIAA1553	KIAA1553	−1.92	−1.65	NM_003472.2	DEK	DEK oncogene (DNA binding)	−1.58	−1.91
NM_001018115.1	FANCD2	Fanconi anemia, complementation group D2	−1.92	−2.39	NM_032015.3	RNF26	ring finger protein 26	−1.58	−1.70
NM_001033.2	RRM1	ribonucleotide reductase M1 polypeptide	−1.92	−1.87	NM_018455.3	CENPN	centromere protein N	−1.57	−2.01
NM_001078174.1	SLC29A1	solute carrier family 29 (nucleoside transporters)	−1.91	−2.19	NM_003091.3	SNRPB	small nuclear ribonucleoprotein polypeptides B and B1	−1.56	−1.52
NM_153485.1	NUP155	nucleoporin 155kDa	−1.91	−1.65	NM_003863.2	DPM2	dolichyl-phosphate mannosyltransferase polypeptide 2	−1.56	−1.77
NM_002482.2	NASP	nuclear autoantigenic sperm protein (histone-binding)	−1.91	−1.96	NM_017916.1	PIH1D1	PIH1 domain containing 1	−1.55	−1.97
NM_176880.4	NR2C2AP	nuclear receptor 2C2-associated protein	−1.90	−1.87	NM_020810.1	TRMT5	tRNA methyltransferase 5 homolog	−1.55	−1.54
NM_021953.2	FOXM1	forkhead box M1	−1.90	−2.28	NM_058219.2	EXOSC6	exosome component 6	−1.55	−1.65
NM_015721.2	GEMIN4	gem (nuclear organelle) associated protein 4	−1.90	−1.54	NM_002882.2	RANBP1	RAN binding protein 1	−1.54	−1.88
NM_018188.2	ATAD3A	ATPase family, AAA domain containing 3A	−1.89	−1.70	NM_007111.3	TFDP1	transcription factor Dp-1	−1.54	−1.51
NM_145060.3	C18orf24	chromosome 18 open reading frame 24	−1.89	−2.45	NM_021709.1	SIVA	CD27-binding (Siva) protein	−1.54	−1.71
NM_018365.1	MNS1	meiosis-specific nuclear structural 1	−1.89	−2.12	NM_015140.2	TTLL12	tubulin tyrosine ligase-like family, member 12	−1.53	−1.61
NM_012310.3	KIF4A	kinesin family member 4A	−1.89	−2.38	NM_022044.2	SDF2L1	stromal cell-derived factor 2-like 1	−1.53	−1.70
NM_015261.2	NCAPD3	non-SMC condensin II complex, subunit D3	−1.88	−1.88	NM_002346.1	LY6E	lymphocyte antigen 6 complex, locus E	−1.52	−1.56
NM_013296.3	GPSM2	G-protein signalling modulator 2	−1.87	−1.63	NM_022490.1	POLR1E	polymerase (RNA) I polypeptide E, 53kDa	−1.52	−1.77
NM_002691.1	POLD1	polymerase (DNA directed), delta 1, catalytic subunit	−1.87	−2.22	NM_032799.4	ZDHHC12	zinc finger, DHHC-type containing 12	−1.51	−1.64
NM_203467.1	PPIL5	peptidylprolyl isomerase (cyclophilin)-like 5	−1.87	−1.79	NM_001017980.2	LOC203547	hypothetical protein	−1.51	−1.54
NM_015201.3	BOP1	block of proliferation 1	−1.87	−2.29	NM_003801.3	GPAA1	glycosylphosphatidylinositol anchor attachment protein 1 homolog	−1.51	−1.89
NM_018353.3	C14orf106	chromosome 14 open reading frame 106	−1.86	−1.54	NM_016594.1	FKBP11	FK506 binding protein 11, 19 kDa	−1.50	−1.94
NM_080626.5	BRI3BP	BRI3 binding protein	−1.84	−1.72	NM_022754.4	SFXN1	sideroflexin 1	−1.50	−1.88

### Modulation of canonical signaling pathways following inhibition of HH signaling

**Figure 3:**
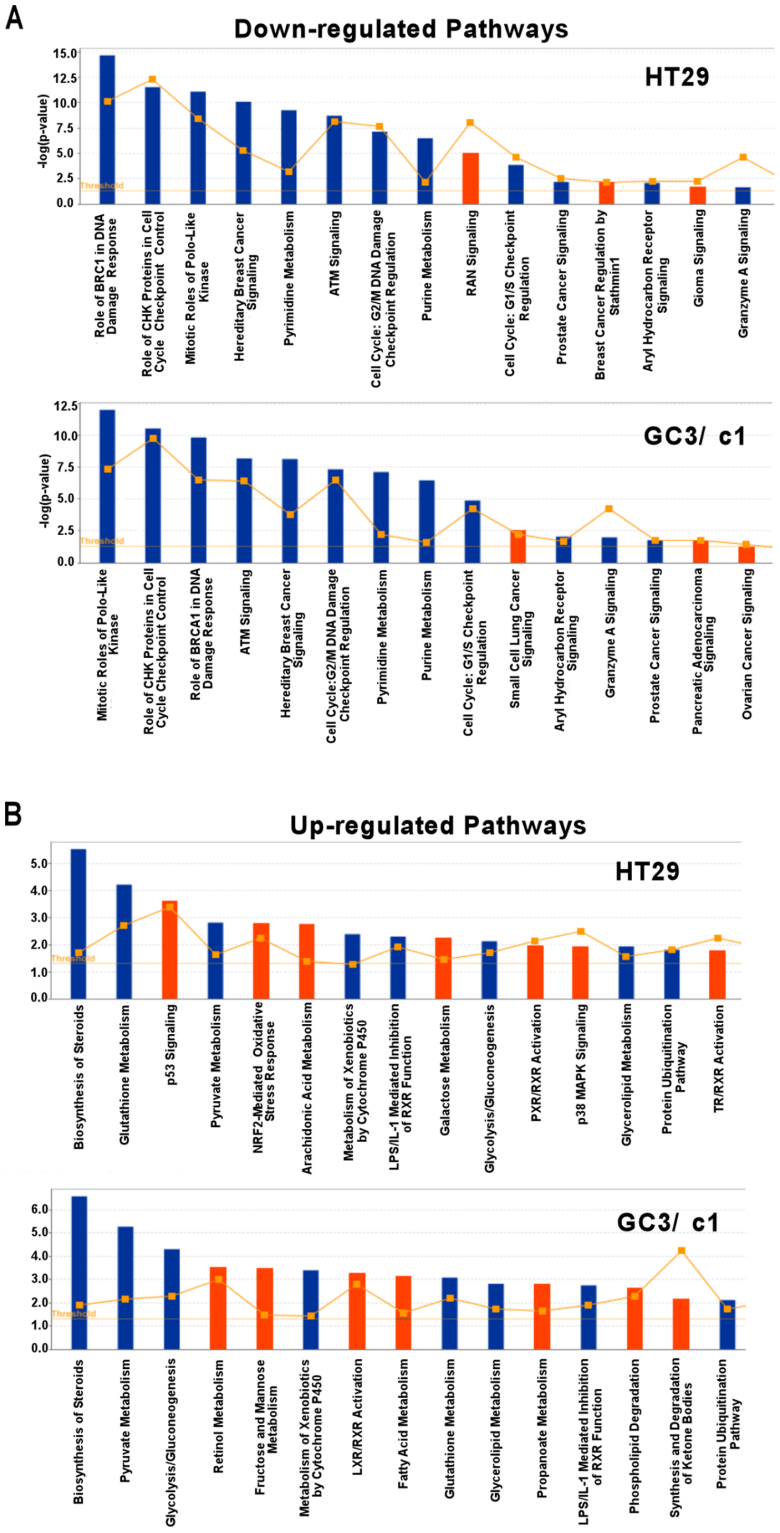
The top 15 canonical signaling pathways influenced by inhibition of GLI1/GLI2 function in HT29 and GC3/c1 cells. The top 15 canonical signaling pathways, determined by IPA, that were significantly up-regulated or down-regulated by GANT61 treatment in HT29 and GC3/c1 cells, are shown. The 1,368 DEGs in HT29 and 1,002 DEGs in GC3/c1 were mapped to the IPA- defined network. The significance p-values that determine the probability that the association between the genes in the dataset and the canonical pathway is by chance alone were calculated by Fisher's exact test, and are expressed as –log (p-value). **A**. Pathways with enriched down-regulated genes. **B**. Pathways with enriched up-regulated genes. Blue: Pathways common to both HT29 and GC3/c1. Red: Pathways unique to either HT29 or GC3/c1. Yellow squares: Ratio of the number of DEGs that map to a specific canonical pathway divided by total number of genes that make up that pathway.

Genes with significant changes in expression following GANT61 treatment were assigned to different canonical signaling pathways and subjected to Ingenuity Pathway Analysis (IPA), where the resulting 1,368 DEGs in HT29 and 1,002 DEGs in GC3/c1 were mapped to networks defined by the IPA database (Figure 3). For the mapped DEGs including both up- and down- regulated genes, the 15 most significantly altered canonical pathways in HT29 demonstrated –log(p-value) ranging from 2.045 to 9.025, and in GC3/c1 from 2.32 to 7.509. Of the 15 pathways involving genes significantly down-regulated, 12 were common to both cell lines. The 3 common pathways with the greatest differential down-regulated expression include genes involved in the DNA damage response, cell cycle checkpoint control, and mitosis. Other pathways down-regulated involved the G1/S and G2/M DNA damage checkpoints, DNA precursor metabolism, and cell signaling involving different pathways including those involved in cancers, which also demonstrate 3 signatures unique to either HT29 or GC3/c1 (Figure 3). Of the 15 pathways involving genes that are the most significantly up-regulated, 8 are common to both HT29 and GC3/c1, and 7 represent unique pathways for each cell line, demonstrating more diversity in patterns of up-regulated gene expression (Figure 3). The up-regulated pathways common to both cell lines include the metabolism-related such as steroids, pyruvate, glycolysis glutathione, or glycerolipid, and not directly related to the control of cellular proliferation.

### Genes that demonstrate differential fold change patterns in GANT61-treated HT29 and GC3/c1 cells

**Figure 4:**
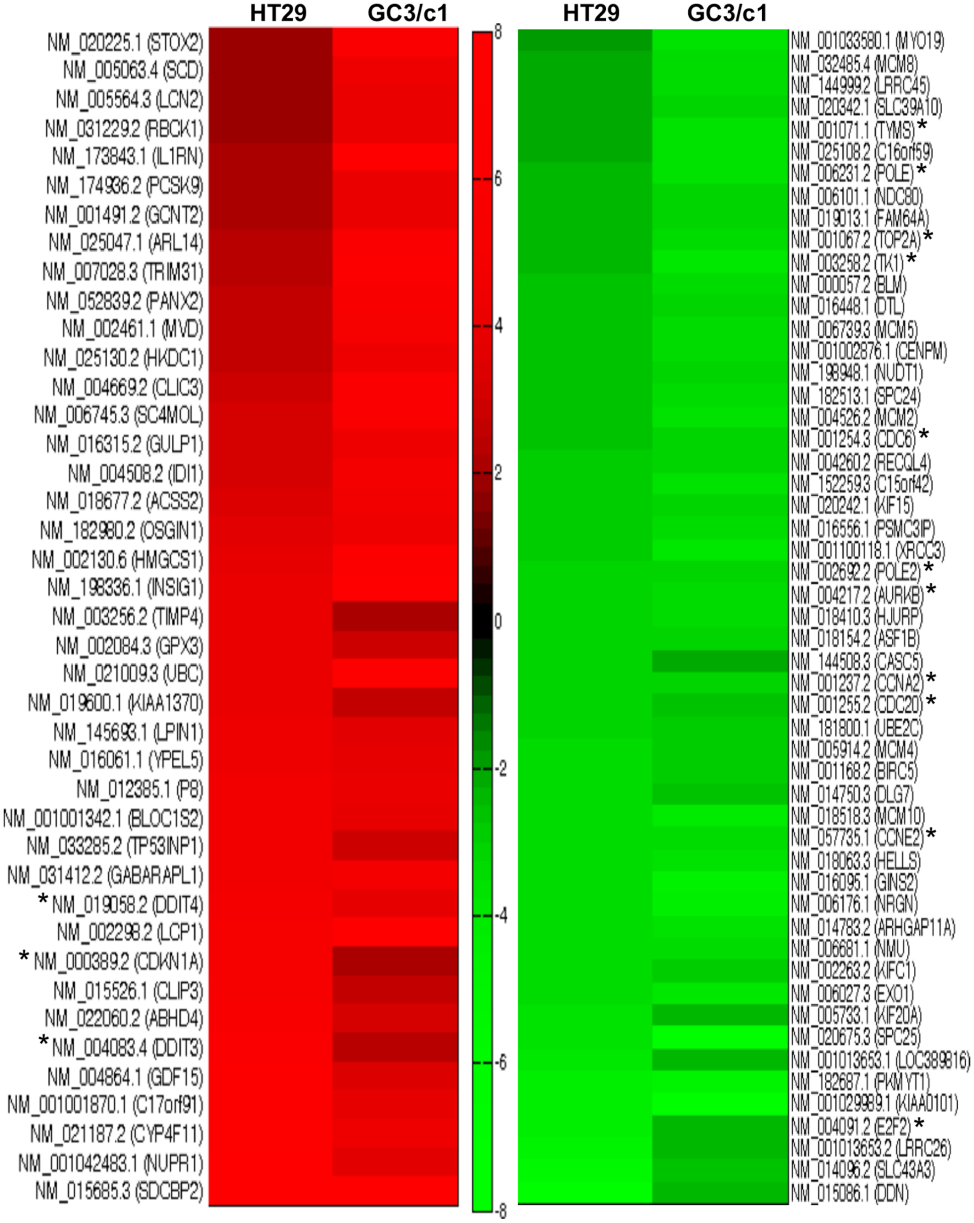
Heat map showing fold change patterns of most highly DEGs in GANT61-treated human colon carcinoma cell lines. The heat map was generated in Matlab (Mathworks), and compares fold change patterns of the most highly DEGs in HT29 and GC3/c1 cells after GANT61 treatment. The most highly DEGs demonstrated a differential expression p-value of p<0.001 between vehicle control (0.2% DMSO) and GANT61-treated cells. Left panel (red): up-regulated genes. Right panel (green): down-regulated genes. Genes denoted with asterisks define those genes with specific roles in G1/S transition, S-phase progression, DNA replication or repair, or regulation of the G2- or M- phase transitions. Fold changes of all down-regulated DEGs and all but one up-regulated DEG are ≤8 (central color spectrum bar).

Fold change patterns of most highly DEGs in HT29 and GC3/c1 were selected, analyzed and displayed in a heat map to evaluate and compare similarity and differences in differential expression between the two cell lines treated with GANT61 (Figure 4). In addition to genes with diverse functions that are not directly related to HH-dependent proliferation, up-regulated genes that influence the G1/S transition and subsequent cell cycle progression, and that are common to both cell lines, include CDKN1A, and the DNA-damage-inducible transcripts 3 and 4 (DDIT3 and DDIT4). A considerably greater number of genes involved in cellular proliferation and cell cycle transition through the G1/S boundary, S-phase progression, and the G2/M transition, were significantly down-regulated in expression, and common to both cell lines. These include CDC6 (involved at G1/S), three genes that drive entry into and passage through S-phase (CCNE2, E2F2) and G2 (CCNA2), genes involved in DNA replication and repair (TYMS, POLE, TOP2A, TK1, POLE2), and two genes that regulate mitosis (AURKB, CDC20; Figure 4, asterisks).

Of the 296 up-regulated genes, in addition to the genes comparably represented in the heat map that include DDIT3 (GADD153) and DDIT4 (REDD1), additional novel DNA damage-inducible transcripts were also identified and include DDIT2 (GADD45G), PPP1R15A (GADD34) and ATF3 (Table 1). TP53INP1, which can regulate cell cycle arrest, and TP53INP2, identified in cell death responses, were also up-regulated. Of the 309 genes significantly down-regulated in response to GANT61, novel genes identified include KIAA0101 (p15[PAF]), Replication Factor C variants 2, 3, 4, 5, CDT1, the E2F transcription factors CDCA4 and TFDP1, MDC1, PCNA, FANCD2, and the genes involved in DNA repair, RAD51C (XRCC3), RAD54B, RAD51 and HELLS (Table 2).

### Differentially expressed genes involved in the G1/S and G2/M transitions

To further evaluate the genes involved in control of cell cycle progression in human colon carcinoma cells following GANT61 treatment, 10 genes involved in the G1/S or G2/M transitions, identified by IPA, were selected for further examination. Genes required at the G1/S boundary for G1/S transition, or for the induction of a G1/S checkpoint following cytostatic signals, include the two cyclin-dependent kinase inhibitors, p21^Cip1^ (CDKN1A) and p15^Ink4B^ (CDKN2B), which were up-regulated by 5.2- and 3.1- fold, 24 hr after GANT61 administration (Table 3). Additional genes required for the G1/S transition that were down-regulated include the E2 transcription factor E2F2 (-4.2-fold), and other critical genes that were down-regulated by 2.1- to 3.2- fold, include CYCLIN E (CCNE2), CDK2 and CDC25A. At G2/M, GANT61 induced down-regulated expression of CCNA2 (CYCLIN A2), CYCLIN B1 (CCNB1), CYCLIN B2 (CCNB2), CDK1 (CDC2), and CDC25C by 2.3- to 3.1- fold (Table 3).

**Table 3:** Gene expression changes from cDNA arrays at G1/S and G2/M.

Cell Cycle Phase	Accession Number	Gene Symbol	Gene Name	Fold Change	
				HT29	GC3/c1
G1/S	NM_000389.2	CDKN1A	cyclin-dependent kinase inhibitor 1A (p21^Cip1^)	+5.21	+2.02
	NM_078487.2	CDKN2B	cyclin-dependent kinase inhibitor 2B (p15^Ink4b^)	+3.13	+2.13
	NM_004091.2	E2F2	E2F transcription factor 2	−4.24	−2.26
	NM_057735.1	CCNE2	cyclin E2	−3.22	−3.24
	NM_001789.2	CDC25A	cell division cycle 25 homolog A	−2.50	−2.45
	NM_001798.2	CDK2	cyclin−dependent kinase 2	−2.07	−1.88
G2/M	NM_001237.2	CCNA2	cyclin A2	−3.11	−3.01
	NM_004701.2	CCNB2	cyclin B2	−2.65	−2.52
	NM_001786.2	CDK1	cyclin-dependent kinase 1	−2.47	−2.63
	NM_031966.2	CCNB1	cyclin B1	−2.36	−2.28
	NM_022809.2	CDC25C	cell division cycle 25 homolog C	−2.27	−2.05

**Figure 5:**
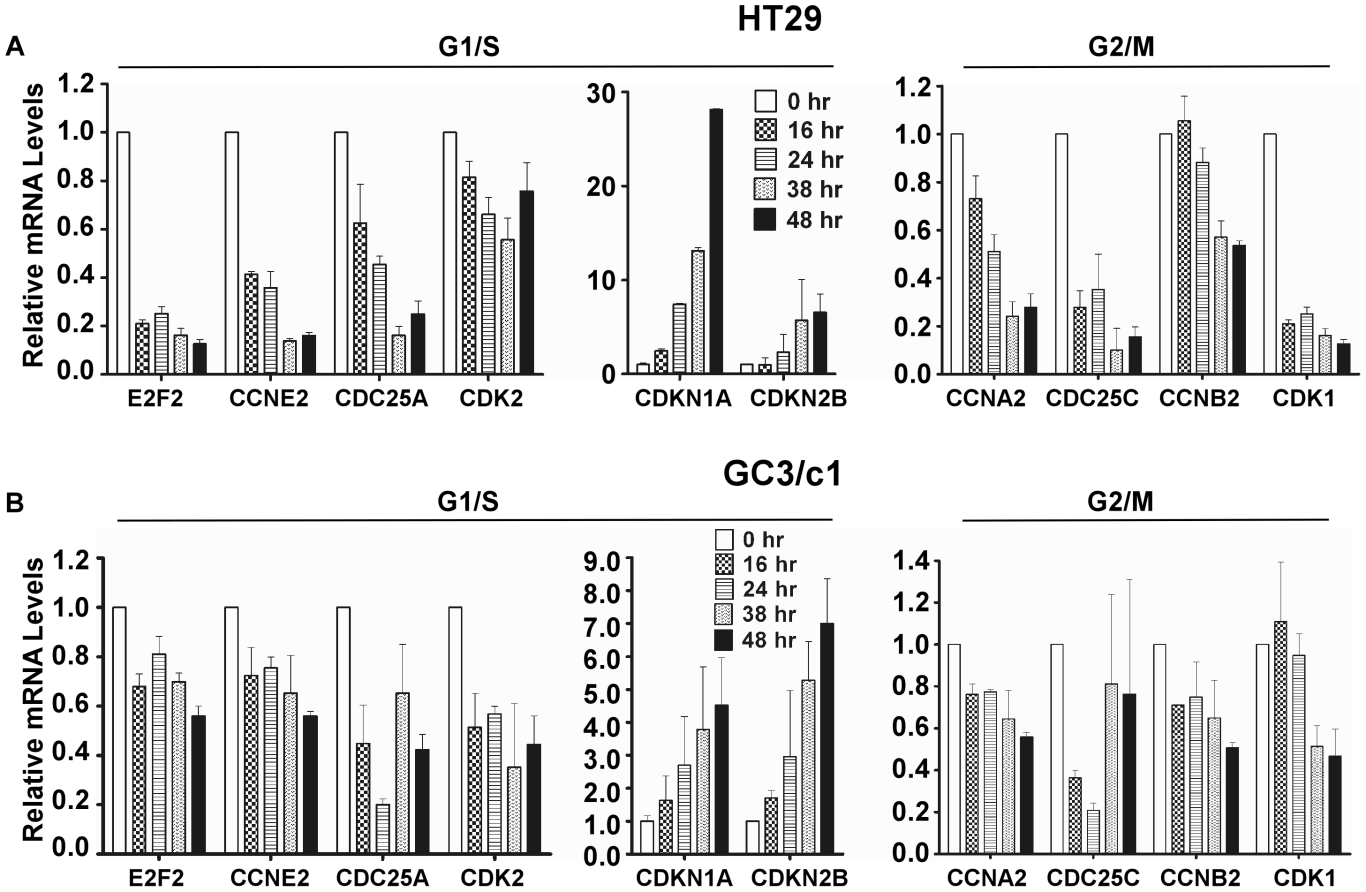
Selected DEGs from cDNA array gene expression profiling analyzed by qRT- PCR in HT29 (A) or GC3/c1 (B). Cells were treated with vehicle alone (0.2% DMSO) or GANT61 (20 µM) for 16 hr, 24 hr, 38 hr, or 48 hr. Total RNA was extracted and qRT-PCR was performed as described in Materials and Methods using the primer sets listed in Table 2. Data represent the mean±SD of 4 determinations, and GAPDH was used to normalize the relative mRNA levels.

To determine the robustness of cDNA microarray gene expression profiling following treatment of HT29 and GC3/c1 cells with GANT61 (20 µM) for 24 hr, qRT-PCR was employed to determine changes in expression of the selected group of 10 DEGs determined from the cDNA microarrays. The genes involved and primers synthesized are shown in Table 4. qRT-PCR was performed on cDNA generated using total RNA independently isolated from GANT61-treated HT29 and GC3/c1 cells for 0 hr, 16 hr, 24 hr, 38 hr and 48 hr after treatment. GAPDH was used to normalize all qRT-PCR data. Genes determined by qRT-PCR included the expression of E2F2, CCNE2, CDC25A and CDK2 at G1/S, which were down-regulated, up-regulation of CDKN1A and CDKN2B at G1/S, and down-regulation of CCNA2, CDC25C, CCNB2, and CDK1 at G2/M (Figure 5). The up-regulated or down-regulated changes in gene expression following GANT61 treatment and determined by cDNA microarray profiling, were confirmed by qRT-PCR (Figure 5).

**Table 4:** Sequences of primers used in quantitative Real-Time PCR.

Accession #	Gene Symbol	Strand	Primer Sequence	Product Size (bp)
NM_078487.2	CDKN2B	Plus	5′-TCTCCGTTGGCCGGAGGTCA-3′	95
		Minus	5′-TGGCAGGGTCTGCGCAGTTG-3′	
NM_004701.2	CCNB2	Plus	5′-CTAACGGCGCCTCGTACGCT-3′	54
		Minus	5′-CAGGGAGGGACGCGGACTGA-3′	
NM_057749.1	CCNE2	Plus	5′-GAGCGGTAGCTGGTCTGGCG-3′	94
		Minus	5′-GGGCTGGGGCTGCTGCTTAG-3′	
NM_001789.2	CDC25A	Plus	5′-CGTGGCTGCCTGCACTCTCA-3′	159
		Minus	5′-GGCTGTCACAGGTGACTGGGG-3′	
NM_001798.3	CDK2	Plus	5′-TTTGCTGAGATGGTGACTCGCCG-3′	159
		Minus	5′-CCGGGCCCACTTGGGGAAAC-3′	
NM_004091.2	E2F2	Plus	5′-CCGGCAGAAGCTGTGTGGGG-3′	97
		Minus	5′-GGCCTCCCTAGGCCCAGCTT-3′	
NM_001237.3	CCNA2	Plus	5′-AAAAGGCAGCGCCCGTCCAA-3′	89
		Minus	5′-CTGCTGCTGCGCTAGACCCC-3′	
NM_005631.3	CDKN1A	Plus	5′-CTGCGCCAGCTGAGGTGTGA-3′	189
		Minus	5′-GCTGCTCGCTGTCCACTGGG-3′	
NM_022809.2	CDC25C	Plus	5′-GTGCATTTAGCTGGGATGACAATGGAA-3′	189
		Minus	5′-GGCCACTTCTGCTCACCTTTGC-3′	
NM_001786.2	CDK1	Plus	5′-ACTGGCTGATTTTGGCCTTGCC-3′	118
		Minus	5′-TGAGTAACGAGCTGACCCCAGCAA-3′	
NM_005269	GLI1	Plus	5′GCCCAGACAGAGGCCCACTC-3′	547
		Minus	5′CTGCAGCCATCCCAACGGCA-3′	
NM_005270	GLI2	Plus	5′-CACCGCTGCTCAAAGAGAA-3′	227
		Minus	5′-TCTCCACGCCACTGTCATT-3′	
NM_000264	PTCH1	Plus	5′-CCACAGAAGCGCTCCTACA-3′	214
		Minus	5′-CTGTAATTTCGCCCCTTCC-3′	

## Discussion

The HH signaling pathway is activated in a variety of human cancers following mutations in genes that regulate canonical HH signaling, including the receptor PTC, and the HH signaling molecule, SMO, and can also be activated via transcriptional up-regulation of the HH ligands (reviewed in [3]). This pathway is becoming of increasing importance due to gaining insight into its prominent role in many developmental processes, and in the maintenance of the malignant phenotype in a wide variety of human cancers, whose growth has been found to be prevented by selective inhibition of constitutive HH pathway activity [14], [35], [36]. Tumors of the brain, prostate, skin, pancreas, and kidney have demonstrated the requirement for HH-GLI signaling, and have responded to inhibition of the HH signaling target molecule SMO by cyclopamine or SMOshRNA [4], [19], [20], [21], [22], [23].

The transcriptional activators in HH signaling comprise members of the GLI family of transcription factors, GLI1 and GLI2, which have both distinct as well as overlapping functions [16]. Activation of the GLI proteins is an intricate process that involves modifications and interactions of a number of positive and negative pathway regulators and is not fully understood [1], [14], [35]. Target genes regulated by the HH signaling pathway differ between tissues and cell types, as well as being influenced by the presence or absence of regulatory factors co-expressed with GLI proteins that eventually determine the transcriptional programs activated by HH signaling [6], [37]. Thus, oncogenic signaling pathways converge on canonical HH signaling at the level of the GLI transcription factors and additionally on target genes downstream of GLI1 and GLI2 to further drive the HH signaling pathway in cellular survival in malignancies [6], [7], [20], [38], [39], [40]. The HH signaling phenotype is therefore significantly influenced and ultimately determined by the co-expression of additional regulatory factors, and hence by the cellular context of gene expression.

HH signaling plays a role in the differentiation program of normal intestinal villi [11], [12], [41], and it has been suggested recently that human colon cancer epithelial cells display a HH-GLI signaling axis in the process of carcinogenesis [24], [25]. Expression of HH-GLI pathway components was consistently demonstrated in an analysis of 40 primary human colon carcinomas and tumors metastatic to the liver [26], consistent with findings of previous investigators [25], [42], [43]. Thus, using qRT-PCR, the expression of GLI1, PTCH1, GLI2 and SHH was determined in all human colon carcinomas examined. The requirement for both GLI1 and GLI2 for sustained proliferation and survival of human colon carcinoma cell lines in vitro, including HT29, was demonstrated using siRNA technology [26]. In addition, knockdown of SMO by SMOshRNA prevented the growth of HT29 cells in SCID mice, while wt HT29 subcutaneous xenografts responded to cyclopamine by reduction in tumor volume [26]. Thus, canonical activation of GLI1 and GLI2 via SMO is important for the survival and proliferation of human colon carcinoma cells in vivo.

In the current study, the function of both GLI1 and GLI2 downstream of SMO was inhibited in the presence of GANT61, a small molecule inhibitor that was identified from a cell-based screen to specifically inhibit GLI1-mediated transcription, but that also inhibited the function of GLI2 [34]. This agent was selected to specifically inhibit the final arbiters of HH signaling, the GLI transcription factors, in elucidation of the downstream target genes that determine HH-dependent proliferation in human colon carcinoma cells. Two cell lines, well characterized in our laboratories, HT29 and GC3/c1, were treated with GANT61 (20 µM) for 24 hr, and the expression of GLI1, GLI2 and PTCH1 mRNA was down-regulated. Further, the effects on cellular proliferation as determined by the distribution of cells within the cell cycle and flow cytometric analysis demonstrated accumulation of cells in G1 following treatment, with a concomitant decrease of cells from the G2/M compartment, and in the case of HT29, also from S-phase, suggesting the induction of a G1/S checkpoint.

**Figure 6:**
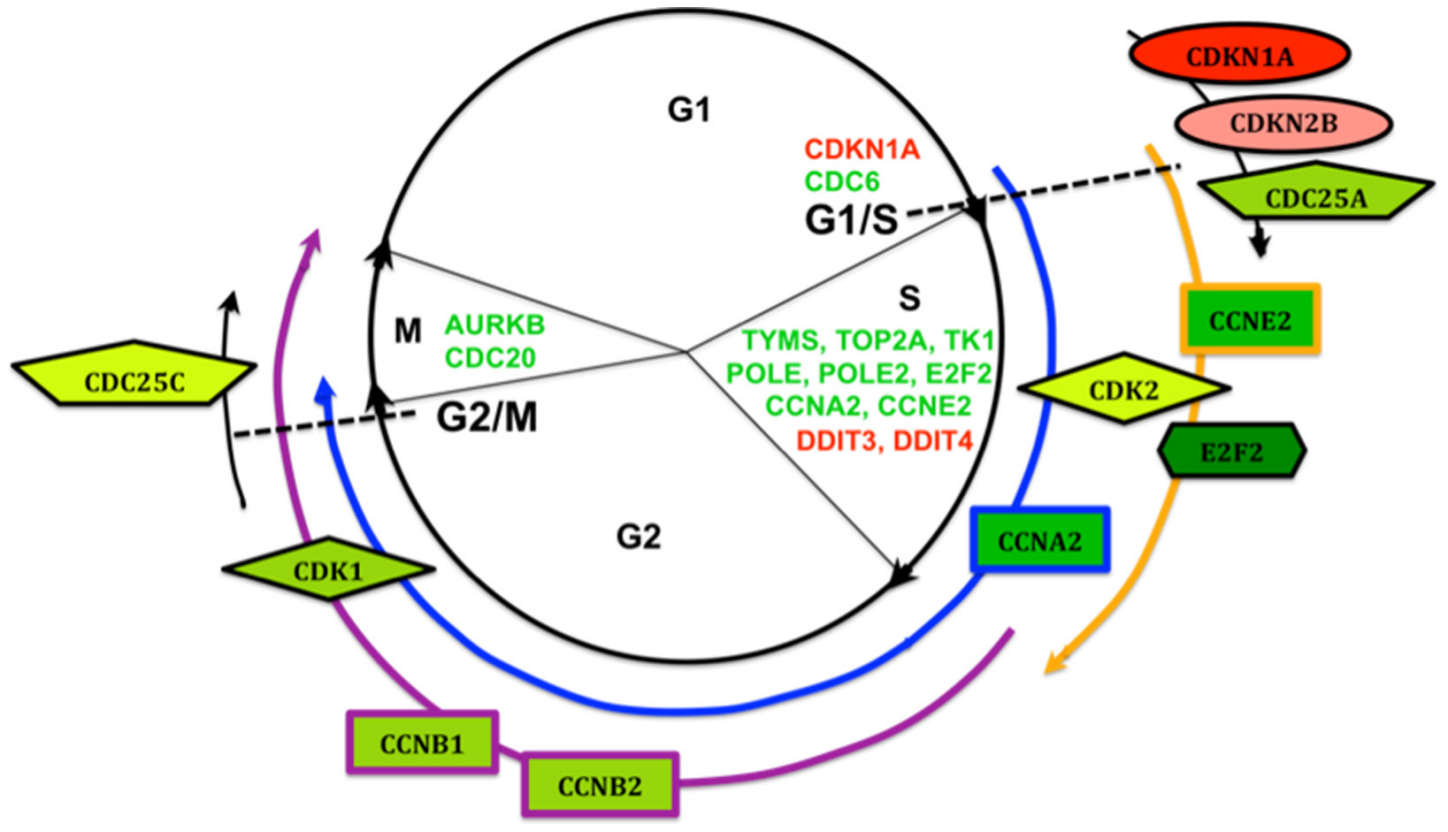
Schematic representation of genes involved in GANT61-induced inhibition of cell cycle progression. From cDNA microarray, heat map, and qRT-PCR analyses, genes involved at different phases of the cell cycle including the G1/S transition, and progression through S- G2- and M- phases, are shown. The genes identified include CDK inhibitors, members of the CDK and CDC families, cyclins, genes involved in DNA replication and repair, and genes that regulate the mitotic spindle, and involve 5 of the 12 common signaling pathways determined by IPA analysis. Red: Up-regulated genes. Green: Down-regulated genes. Light shade→dark shade, increasing differential expression.

HT29 and GC3/c1 cells were subsequently treated with GANT61 (20 µM) for 24 hr, RNA was extracted, and changes in gene expression were determined by Illumina cDNA microarray profiling. Following statistical analyses, 1,368 genes in HT29 and 1,002 genes in GC3/c1, were determined to be significantly modulated by GANT61 treatment (FC>1.5; p<0.001). For genes that were up-regulated in expression, 296 genes were common to both cell lines, and for down-regulated genes, 309 genes were common to both cell lines. The blockade of cells at the G1/S boundary is evidenced by up-regulated expression of p21^Cip1^ and p15^Ink4b^ that in part regulate the G1/S transition. p15^Ink4b^ is a member of the Ink4 family of CDK inhibitors, is induced in response to cytostatic signals [32], [44], and complexes with CYCLIN D/CDK4 or CYCLIN D/CDK6 to mediate G1-phase arrest at the G1/S transition in certain systems [30], [32]. p21^Cip1^ can bind a broad range of cyclin-CDK complexes, with a preference for those containing CDK2 (reviewed in [31], [45], and during a normal cell cycle, facilitates active cyclin-CDK complex formation to promote cell proliferation. However when overexpressed, p21^Cip1^ forms an inhibitory complex with CYCLIN E/CDK2, leading to G1- and consequently S- phase arrest, thereby forming the G1/S checkpoint [46], [47]. The scheduled timing of expression of CYCLINS E, A and B that drive cell cycle progression, is reflected in the major changes in the phases of the cell cycle. CYCLIN E is expressed at maximal levels in cells undergoing the G1 to S transition, declining during S-phase progression, such that G2/M cells are CYCLIN E-negative (reviewed in [30]). CYCLIN A is expressed in late G1, demonstrates pronounced expression during S-phase, and increases as the cells advance towards G2, with degradation in early mitosis; B type cyclins begin to be expressed in late S-phase, and drive the cells though G2- and M- phases of the cell cycle [30]. CDK2 controls the G1/S transition by complexing with CYCLIN E, and is activated by CDC25A, which dephosphorylates CDK2 [48]. CDC2 controls cellular entry into mitosis at the G2/M transition, thereby forming complexes with CYCLINS A and B, is activated by CDC25C, and is down-regulated in late M-phase [29]. All of these genes are down-regulated in expression in response to the inhibition of GLI1/GLI2 function (Table 2). Thus, signals elicited to promote cellular accumulation at G1/S lead to the repression of genes that regulate further cell cycle progression, and p21^Cip1^ expression has been shown to deplete the expression of genes that regulate DNA replication and repair, and mitosis [49], [50], [51]. In the current study these genes include CDC6 (active at the G1/S transition and essential for the initiation of DNA replication), TYMS, TOP2A, TK1, POLE and POLE2 (S-phase), and AURKB and CDC20 (mitosis [52], [53]), determined by heat map analysis. A schematic representation of the genes involved in GANT61-induced inhibition of cell cycle progression at G1/S, S-phase progression, and regulation during G2- and M-phases, identified from cDNA microarrays, heat map analysis, and by qRT-PCR, is shown in Figure 6, and involves 5 of the 12 common signaling pathways determined by IPA analysis.

Comprehensive cDNA microarray gene profiling analysis of genes that determine the HH signaling phenotype has been conducted only in non-cancer cell models. In these systems, GLI activation has been stimulated by EGF treatment [6], stable GLI1 or HA-RAS expression [7], or expression of constitutively activated GLI2 [16]. In these studies, CYCLIN D [6], [7], GADD153 [7], CDKN2B, CDKN1A, CDK2, PCNA, TOP2A, CCNB1, XRCC1 [16], have been identified as genes activated downstream of GLI. In GANT61-treated human colon carcinoma cells, novel DNA damage-inducible transcripts DDIT3 (GADD153), DDIT4 (REDD1), DDIT2 (GADD45G), PPP1R15A (GADD34) and ATF3 were significantly up-regulated concomitant with the arrest of cells at G1/S. TP53INP1, involved in cell cycle regulation, and TP53INP2, linked to cell death responses, were also up-regulated. Additional novel genes involved in S-phase progression and DNA damage response that were significantly down-regulated include KIAA0101 (p15[PAF]), Replication Factor C variants 2, 3, 4, 5, CDT1, the E2F transcription factors CDCA4 and TFDP1, MDC1, PCNA, FANCD2, and the genes involved in DNA repair, RAD51C (XRCC3), RAD54B, RAD51 and HELLS.

In summary, we have compared gene expression profiles in two human colon carcinoma cell lines after targeting the function of the transcriptional regulators of HH signaling, GLI1 and GLI2, using the small molecule inhibitor GANT61. Data are consistent with accumulation of cells at the G1/S boundary, as evidenced from flow cytometric analysis, cDNA microarray gene profiling, and qRT- PCR. GANT61-treated cells demonstrated up-regulated expression of the CDK inhibitors p21^Cip1^ and p15^Ink4b^ that function at the G1/S boundary, and down-regulated expression of additional key genes that determine the G1/S transition, initiation of DNA replication, S-phase progression, DNA repair, and subsequent transition through the G2/M phases. Inhibition of the transcriptional regulation of HH signaling in human colon carcinoma cells therefore directly involves genes that regulate cell cycle transition through G1/S, cell cycle progression, and proliferation, and genes involved in stress-induced and DNA damage responses.

## Materials and Methods

### Human colon carcinoma cell lines

HT29 was purchased from ATCC (Manassas, VA), while GC3/c1 was established in culture by our group from a human colon adenocarcinoma xenograft model [54]; both cell lines express mutant p53 alleles. Cell lines were maintained in the presence of folate-free RPMI 1640 medium containing 10% dFBS and 80 nM [6RS] 5-methyltetrahydrofolate.

### Flow cytometric analysis

HT29 and GC3/c1 cells were plated at a density of 100,000 cells/well in six-well plates. After overnight attachment, cells were treated with GANT61 (20µM; Enzo Life Sciences, Germany) or vehicle control (DMSO, 0.2%), in duplicate, for 24 hr, followed by washing ×1 with PBS, trypsinization, and centrifugation. Cells were fixed with 70% ethanol at RT, 20–30 min, stored at −20°C overnight, centrifuged for 5 min at 200×g to remove ethanol, and washed ×1 in PBS. The cells were resuspended in PBS, and low molecular weight DNA was extracted using DNA extraction buffer (0.2M Na_2_HPO_4_ and 0.1M citric acid, pH 7.8) for 5 min. The extracted DNA was centrifuged and resuspended in DNA staining solution containing propidium iodide (50 µg) and DNAse-free RNAse (2 mg), and allowed to incubate for 30 min in the dark at RT. Distribution of cells throughout the cell cycle was analyzed using a FACSCalibur flow cytometer, and data were analyzed using CellQuest software.

### RNA isolation

Cells were seeded at 7×10^6^ cells/10 cm plate overnight at ≈60% confluency, and subsequently treated with either vehicle control (0.2% DMSO) or GANT61 (20 µM), in duplicate, for 24 hr. Cells were subsequently harvested and RNA was isolated using the RNeasy® Mini Kit (Qiagen, Valencia, CA) following the manufacturer's protocol. Integrity of the RNA was determined by spectrophotometry and electrophoresis.

### cDNA microarray analysis

RNA (250 ng) was reverse transcribed into cRNA and biotin-UTP labeled using the Illumina® TotalPrep RNA Amplification Kit (Ambion, Applied Biosystems, Foster City, CA) according to the manufacturer's protocol. cRNA was quantified using a nanodrop spectrophotometer, and the cRNA quality (size distribution) further analyzed on a 1% agarose gel. Biotinylated cRNAs were hybridized to the Illumina Human-ref8 V3.0 BeadChip (Illumina, San Diego, CA) that represents 18,401 genes, using standard protocols. The arrays were washed and subsequently scanned using an Illumina BeadArray Reader.

Raw signal intensities of gene expression data were processed and analyzed using GenomeStudio (Illumina), with background subtraction and average normalization performed on the average signal intensities, and p-values were calculated. Raw data were exported from GenomeStudio into Excel (available as Data S1 and Data S2), and the fold change in gene expression calculated, by dividing the average gene expression signal intensity of treated samples (GANT61) with that of the vehicle control (DSMO). Differential gene expression (DEG) analysis between control and GANT61-treated samples, was subsequently conducted using the Illumina customer model, which applies multiple testing corrections that determines the false discovery rate (FDR; [55]. Thus, FDR-adjusted differential scores and p-values for each gene/probe set between treated and control samples were generated. Genes with a FDR-adjusted p-value of p<0.001 and fold change ≥1.5 were considered to be DEGs and were subjected to Venn analysis and Ingenuity Pathway analysis.

These differentially expressed genes were, uploaded and mapped to the library of canonical pathways of the Ingenuity Pathways Analysis (IPA) database for pathway analysis (Ingenuity® Systems, http://www.ingenuity.com, Mountain View, CA). The mapped datasets containing either up-regulated or down-regulated genes in HT29 or GC3/c1 cells with corresponding expression values were subjected to core analysis that includes overall canonical pathway enrichment analysis. The significance of enrichment of genes mapped to different canonical pathways was calculated by the Fischer's exact test (p-value) to determine the probability that the association between the genes and the canonical pathway could be explained by chance alone. Further, the ratio between the number of identified genes in a particular pathway and total number of genes that make up that pathway provides an estimation of the extent of pathway involvement. The enriched canonical pathways were ranked by −log (p-value) as shown in a histogram of pathway vs. –log (p-value). Datasets containing both up-regulated and down-regulated genes were also analyzed in selected pathways pertaining to cell cycle progression at G1/S and G2/M.

Fold change patterns of most highly DEGs in HT29 and GC3/c1 were compared by Heat map analysis using Matlab (Mathworks) software. DEGs analyzed and displayed in the heat map demonstrated a differential expression p-value of p<0.001 between control and GANT61 treated cells in both cell lines.

### Quantitative Real-Time PCR (qRT-PCR)

The expression levels of selected genes identified by cDNA microarray expression profiling at 24 hr following GANT61 treatment, were validated by qRT-PCR. Thus, HT29 or GC3/c1 cells were either untreated (vehicle control, 0.2% DMSO), or treated with GANT61 (20 µM) for 0 hr, 16 hr, 24 hr, 38 hr or 48 hr at 37°C, dissolved in DMSO-containing medium. Total RNA (1 µg) was employed to prepare cDNA via reverse transcription using the iScript Select cDNA Synthesis Kit (Biorad) Reverse Transcription System according to manufacturers instructions and analyzed using an Applied Biosystems 7500 PCR Detection System (Applied Biosystems Inc.). All amplifications were primed by pairs of chemically synthesized 18- to 24- mer oligonucleotides designed using freely available primer design software (Primer-BLAST, NCBI) to generate target amplicons of 50–547 bp. All reactions were performed in a final volume of 15 µl. qRT-PCR reaction conditions were as follows: activation at 95°C for 10 min with 40 cycles of denaturation at 95°C for 15 s, primer annealing and extension at 60°C for 1 min and ramping back to 95°C. Melt curve analysis of all samples was routinely performed to ascertain that only the expected products had been generated. A fluorescence reading determined the extent of amplification at the end of each cycle. mRNA expression levels of target genes were normalized to the expression of glyceraldehyde phosphate dehydrogenase (GAPDH) and quantified using the comparative CT method [56]. Q-RT-PCR for each gene was determined in duplicate, and each experiment was repeated at least twice.

## Supporting Information

Data S1 HT29 Raw Microarray Data.
(11.39 MB XLS)

Data S2 GC3/c1 Raw Microarray Data.
(11.06 MB XLS)

## References

[1] Lum L, Beachy PA (2004). The Hedgehog response network: sensors, switches, and routers.. Science.

[2] Hooper JE, Scott MP (2005). Communicating with Hedgehogs.. Nat Rev Mol Cell Biol.

[3] Katoh Y, Katoh M (2009). Hedgehog target genes: mechanisms of carcinogenesis induced by aberrant hedgehog signaling activation.. Curr Mol Med.

[4] Ruiz i Altaba A, Mas C, Stecca B (2007). The Gli code: an information nexus regulating cell fate, stemness and cancer.. Trends Cell Biol.

[5] Yu M, Gipp J, Yoon JW, Iannaccone P, Walterhouse D (2009). Sonic hedgehog-responsive genes in the fetal prostate.. J Biol Chem.

[6] Kasper M, Schnidar H, Neill GW, Hanneder M, Klingler S (2006). Selective modulation of Hedgehog/GLI target gene expression by epidermal growth factor signaling in human keratinocytes.. Mol Cell Biol.

[7] Yoon JW, Kita Y, Frank DJ, Majewski RR, Konicek BA (2002). Gene expression profiling leads to identification of GLI1-binding elements in target genes and a role for multiple downstream pathways in GLI1-induced cell transformation.. J Biol Chem.

[8] Katoh Y, Katoh M (2008). Integrative genomic analyses on GLI2: mechanism of Hedgehog priming through basal GLI2 expression, and interaction map of stem cell signaling network with P53.. Int J Oncol.

[9] Regl G, Kasper M, Schnidar H, Eichberger T, Neill GW (2004). The zinc-finger transcription factor GLI2 antagonizes contact inhibition and differentiation of human epidermal cells.. Oncogene.

[10] Varnat F, Zacchetti G, Ruiz i Altaba A. Hedgehog pathway activity is required for the lethality and intestinal phenotypes of mice with hyperactive Wnt signaling.. Mech Dev.

[11] Alinger B, Kiesslich T, Datz C, Aberger F, Strasser F (2009). Hedgehog signaling is involved in differentiation of normal colonic tissue rather than in tumor proliferation.. Virchows Arch.

[12] van den Brink GR (2004). Linking pathways in colorectal cancer.. Nat Genet.

[13] Katoh Y, Katoh M (2006). Hedgehog signaling pathway and gastrointestinal stem cell signaling network (review).. Int J Mol Med.

[14] Kasper M, Regl G, Frischauf AM, Aberger F (2006). GLI transcription factors: mediators of oncogenic Hedgehog signalling.. Eur J Cancer.

[15] Thiyagarajan S, Bhatia N, Reagan-Shaw S, Cozma D, Thomas-Tikhonenko A (2007). Role of GLI2 transcription factor in growth and tumorigenicity of prostate cells.. Cancer Res.

[16] Eichberger T, Sander V, Schnidar H, Regl G, Kasper M (2006). Overlapping and distinct transcriptional regulator properties of the GLI1 and GLI2 oncogenes.. Genomics.

[17] Ruiz i Altaba A (1999). Gli proteins encode context-dependent positive and negative functions: implications for development and disease.. Development.

[18] Nguyen V, Chokas AL, Stecca B, Ruiz i Altaba A (2005). Cooperative requirement of the Gli proteins in neurogenesis.. Development.

[19] Sanchez P, Hernandez AM, Stecca B, Kahler AJ, DeGueme AM (2004). Inhibition of prostate cancer proliferation by interference with SONIC HEDGEHOG-GLI1 signaling.. Proc Natl Acad Sci U S A.

[20] Stecca B, Mas C, Clement V, Zbinden M, Correa R (2007). Melanomas require HEDGEHOG-GLI signaling regulated by interactions between GLI1 and the RAS-MEK/AKT pathways.. Proc Natl Acad Sci U S A.

[21] Feldmann G, Dhara S, Fendrich V, Bedja D, Beaty R (2007). Blockade of hedgehog signaling inhibits pancreatic cancer invasion and metastases: a new paradigm for combination therapy in solid cancers.. Cancer Res.

[22] Sarangi A, Valadez JG, Rush S, Abel TW, Thompson RC (2009). Targeted inhibition of the Hedgehog pathway in established malignant glioma xenografts enhances survival.. Oncogene.

[23] Dormoy V, Danilin S, Lindner V, Thomas L, Rothhut S (2009). The sonic hedgehog signaling pathway is reactivated in human renal cell carcinoma and plays orchestral role in tumor growth.. Mol Cancer.

[24] Yoshikawa K, Shimada M, Miyamoto H, Higashijima J, Miyatani T (2009). Sonic hedgehog relates to colorectal carcinogenesis.. J Gastroenterol.

[25] Bian YH, Huang SH, Yang L, Ma XL, Xie JW (2007). Sonic hedgehog-Gli1 pathway in colorectal adenocarcinomas.. World J Gastroenterol.

[26] Varnat F, Duquet A, Malerba M, Zbinden M, Mas C (2009). Human colon cancer epithelial cells harbour active HEDGEHOG-GLI signalling that is essential for tumour growth, recurrence, metastasis and stem cell survival and expansion.. EMBO Mol Med.

[27] Nurse P (2000). A long twentieth century of the cell cycle and beyond.. Cell.

[28] Berger JH, Bardeesy N (2007). Modeling INK4/ARF tumor suppression in the mouse.. Curr Mol Med.

[29] Stark GR, Taylor WR (2006). Control of the G2/M transition.. Mol Biotechnol.

[30] McDonald ER, El-Deiry WS (2000). Cell cycle control as a basis for cancer drug development (Review).. Int J Oncol.

[31] Harper JW, Elledge SJ, Keyomarsi K, Dynlacht B, Tsai LH (1995). Inhibition of cyclin-dependent kinases by p21.. Mol Biol Cell.

[32] Choi S, Kim TW, Singh SV (2009). Ginsenoside Rh2-mediated G1 phase cell cycle arrest in human breast cancer cells is caused by p15 Ink4B and p27 Kip1-dependent inhibition of cyclin-dependent kinases.. Pharm Res.

[33] Polager S, Kalma Y, Berkovich E, Ginsberg D (2002). E2Fs up-regulate expression of genes involved in DNA replication, DNA repair and mitosis.. Oncogene.

[34] Lauth M, Bergstrom A, Shimokawa T, Toftgard R (2007). Inhibition of GLI-mediated transcription and tumor cell growth by small-molecule antagonists.. Proc Natl Acad Sci U S A.

[35] Ingham PW, McMahon AP (2001). Hedgehog signaling in animal development: paradigms and principles.. Genes Dev.

[36] Ruiz i Altaba A, Sanchez P, Dahmane N (2002). Gli and hedgehog in cancer: tumours, embryos and stem cells.. Nat Rev Cancer.

[37] Ingham PW, Placzek M (2006). Orchestrating ontogenesis: variations on a theme by sonic hedgehog.. Nat Rev Genet.

[38] Riobo NA, Lu K, Ai X, Haines GM, Emerson CP (2006). Phosphoinositide 3-kinase and Akt are essential for Sonic Hedgehog signaling.. Proc Natl Acad Sci U S A.

[39] Riobo NA, Haines GM, Emerson CP (2006). Protein kinase C-delta and mitogen-activated protein/extracellular signal-regulated kinase-1 control GLI activation in hedgehog signaling.. Cancer Res.

[40] Schnidar H, Eberl M, Klingler S, Mangelberger D, Kasper M (2009). Epidermal growth factor receptor signaling synergizes with Hedgehog/GLI in oncogenic transformation via activation of the MEK/ERK/JUN pathway.. Cancer Res.

[41] Varnat F, Zacchetti G, Ruiz IAA (2009). Hedgehog pathway activity is required for the lethality and intestinal phenotypes of mice with hyperactive Wnt signaling.. Mech Dev.

[42] Monzo M, Moreno I, Artells R, Ibeas R, Navarro A (2006). Sonic hedgehog mRNA expression by real-time quantitative PCR in normal and tumor tissues from colorectal cancer patients.. Cancer Lett.

[43] Oniscu A, James RM, Morris RG, Bader S, Malcomson RD (2004). Expression of Sonic hedgehog pathway genes is altered in colonic neoplasia.. J Pathol.

[44] Koyama M, Matsuzaki Y, Yogosawa S, Hitomi T, Kawanaka M (2007). ZD1839 induces p15INK4b and causes G1 arrest by inhibiting the mitogen-activated protein kinase/extracellular signal-regulated kinase pathway.. Mol Cancer Ther.

[45] Gartel AL, Tyner AL (2002). The role of the cyclin-dependent kinase inhibitor p21 in apoptosis.. Mol Cancer Ther.

[46] Niculescu AB, Chen X, Smeets M, Hengst L, Prives C (1998). Effects of p21(Cip1/Waf1) at both the G1/S and the G2/M cell cycle transitions: pRb is a critical determinant in blocking DNA replication and in preventing endoreduplication.. Mol Cell Biol.

[47] Ogryzko VV, Wong P, Howard BH (1997). WAF1 retards S-phase progression primarily by inhibition of cyclin-dependent kinases.. Mol Cell Biol.

[48] Sandhu C, Donovan J, Bhattacharya N, Stampfer M, Worland P (2000). Reduction of Cdc25A contributes to cyclin E1-Cdk2 inhibition at senescence in human mammary epithelial cells.. Oncogene.

[49] Abbas T, Dutta A (2009). p21 in cancer: intricate networks and multiple activities.. Nat Rev Cancer.

[50] Chang BD, Watanabe K, Broude EV, Fang J, Poole JC (2000). Effects of p21Waf1/Cip1/Sdi1 on cellular gene expression: implications for carcinogenesis, senescence, and age-related diseases.. Proc Natl Acad Sci U S A.

[51] Chang BD, Broude EV, Fang J, Kalinichenko TV, Abdryashitov R (2000). p21Waf1/Cip1/Sdi1-induced growth arrest is associated with depletion of mitosis-control proteins and leads to abnormal mitosis and endoreduplication in recovering cells.. Oncogene.

[52] Keen N, Taylor S (2009). Mitotic drivers–inhibitors of the Aurora B Kinase.. Cancer Metastasis Rev.

[53] Ge S, Skaar JR, Pagano M (2009). APC/C- and Mad2-mediated degradation of Cdc20 during spindle checkpoint activation.. Cell Cycle.

[54] Tillman DM, Izeradjene K, Szucs KS, Douglas L, Houghton JA (2003). Rottlerin sensitizes colon carcinoma cells to tumor necrosis factor-related apoptosis-inducing ligand-induced apoptosis via uncoupling of the mitochondria independent of protein kinase C.. Cancer Res.

[55] Reiner A, Yekutieli D, Benjamini Y (2003). Identifying differentially expressed genes using false discovery rate controlling procedures.. Bioinformatics.

[56] Livak KJ, Schmittgen TD (2001). Analysis of relative gene expression data using real-time quantitative PCR and the 2(-Delta Delta C(T)) Method.. Methods.

